# A Concise Route to Water-Soluble 2,6-Disubstituted BODIPY-Carbohydrate Fluorophores by Direct Ferrier-Type C-Glycosylation

**DOI:** 10.1021/acs.joc.1c00413

**Published:** 2021-06-22

**Authors:** Ana M. Gómez, Clara Uriel, Ainhoa Oliden-Sánchez, Jorge Bañuelos, Inmaculada Garcia-Moreno, J. Cristobal López

**Affiliations:** † Instituto de Química Orgánica General, IQOG-CSIC, Juan de la Cierva 3, 28006 Madrid, Spain; ‡ Departamento de Química Física, Universidad del Pais Vasco, UPV-EHU, Apartado 644, 48080 Bilbao, Spain; § Instituto de Química-Física Rocasolano, CSIC, Serrano 119, 28006 Madrid, Spain

## Abstract

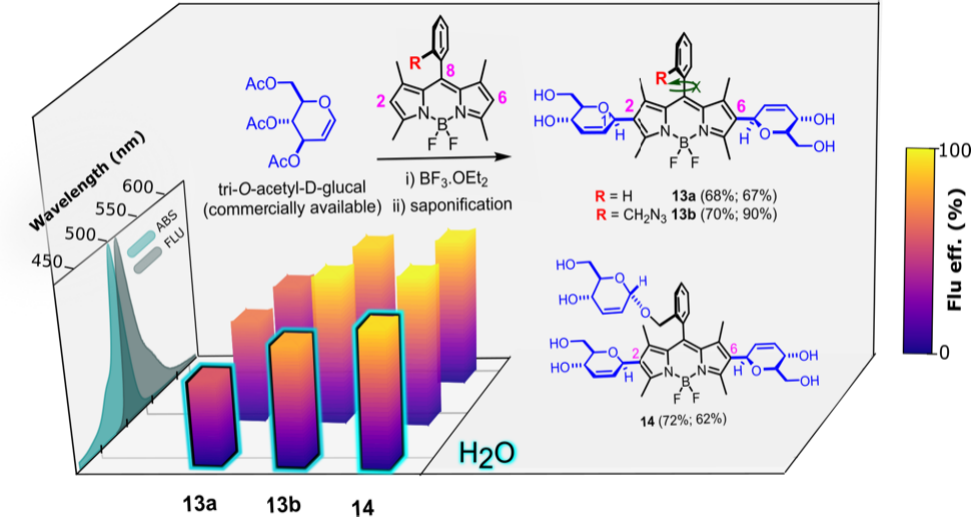

Novel, linker-free, BODIPY-carbohydrate derivatives containing sugar residues at positions C2 and C6 are efficiently obtained by, hitherto unreported, Ferrier-type *C*-glycosylation of 8-aryl-1,3,5,7-tetramethyl BODIPYs with commercially available tri-*O*-acetyl-d-glucal followed by saponification. This transformation, which involves the electrophilic aromatic substitution (S_E_Ar) of the dipyrrin framework with an allylic oxocarbenium ion, provides easy access to BODIPY-carbohydrate hybrids with excellent photophysical properties and a weaker tendency to aggregate in concentrated water solutions.

The burgeoning interest in fluorescence imaging techniques as non-invasive, highly sensitive, and operationally simple ways to visualize biological processes has spurred the development of biocompatible, water-soluble fluorophores.^1^ In this context, the consideration of boron dipyrromethene difluoride (BODIPY) dyes, e.g., **1** (Figure 1), has amply surpassed that of the traditionally studied fluorescein, cyanine, and rhodamine fluorophores.^2^ Thus, among other fluorophores, BODIPYs excel in their remarkable properties, including strong absorption, high molar absorption coefficients and fluorescence quantum yields, good chemical and photochemical stability, and low toxicity.^3^ Nevertheless, arguably, their main appeal might be their ability to fine-tune their spectroscopic and photophysical properties by postfunctional modifications of the dipyrromethene core.^4^

**Figure 1:**
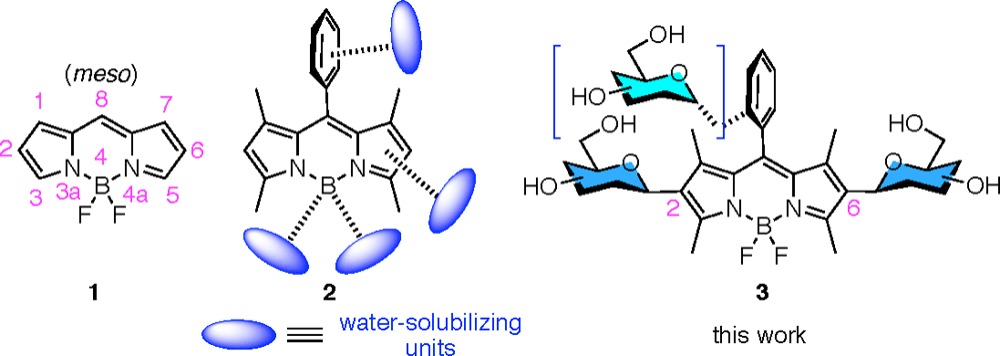
BODIPY (**1**, IUPAC numbering), BODIPY equipped for solubilization in water (**2**), and water-soluble, linker-free, 2,6-substituted glyco-BODIPYs (**3**).

In this context, a variety of postfunctionalization studies have emerged to improve water solubility and minimize aggregation-induced quenching of BODIPY fluorophores.^5^ These studies have involved the peripheral incorporation of charged anionic, cationic, and zwitterionic functionalities, the grafting of the BODIPY to neutral hydrophilic compounds, or combinations thereof, e.g., **2** (Figure 1).^6−8^ On the contrary, the introduction of bulky substituents into the fluorophore core, or at the apical position, has been used to prevent aggregation, a phenomenon known to lower the quantum yield of fluorophores.^9^

Among the neutral hydrophilic derivatives employed for solubilization of BODIPYs, carbohydrates have received more consideration. This attention is, very likely, motivated by the fact that carbohydrates, in addition to water solubility, might provide biocompatibility and enhanced targeting ability to the ensuing glyco-BODIPYs.^10^

In general, carbohydrates have been incorporated into the periphery of the BODIPY core, frequently by being attached to alkyl or aryl substituents located at the *meso* (C8) position,^11^ or at the boron atom,^12^ normally through a linker, in transformations that generally involve copper(I)-catalyzed azide–alkyne cycloadditions (CuAAC).^13^ On the contrary, scarce examples of *O*-glycosylation reactions, the most common being the glycosyl coupling method,^14^ have been reported for the assembly of carbohydrates with BODIPYs.^15^

According to these precedents, we envisioned that it would be of interest to develop a *C*-glycosylation protocol^16^ that could engage positions C2 and C6 of the BODIPY core, in commonly used 1,3,5,7-tetramethyl BODIPYs. Such a method could provide direct access to “linker-free”, nonhydrolyzable, water-soluble bis-*C*-glycosidic BODIPYs, e.g., **3** (Figure 1).

Thus, even though positions C2 and C6 of the boraindacene core are prone to experiencing electrophilic aromatic substitution (S_E_Ar) reactions,^4^ to the best of our knowledge, no *C*-glycosylation reaction has been reported to date. In this context, we had already observed the reluctance of the BODIPY core to undergo such a reaction in glycosylations of hydroxyl-containing BODIPYs with common glycosyl donors, where no sign of *C*-glycosylation adducts had been detected.^15b,15d^ Furthermore, in the course of this work, the attempted reaction of 8-aryl-1,3,5,7-tetramethyl BODIPYs with glycosyl trichloroacetimidate donors failed to provide any *C*-glycosylated BODIPYs. We, therefore, reasoned that compared to a classical glycosyl oxonium ion, e.g., **4** (Figure 2), arising from a standard glycosyl donor, a more stabilized and less sterically encumbered, allylic oxocarbenium ion, i.e., **5** (Figure 2),^17,18^ might be able to glycosylate the 4-bora-3a,4a-diaza-*s*-indacene skeleton. Under such premises, we decided to test the electrophilic Ferrier-type *C*-glycosylation reaction (which involves allylic cation **5**) of 8-aryl and 8-methyl 1,3,5,7-tetramethyl BODIPYs **6** and **7**, respectively (Figure 2).

**Figure 2:**
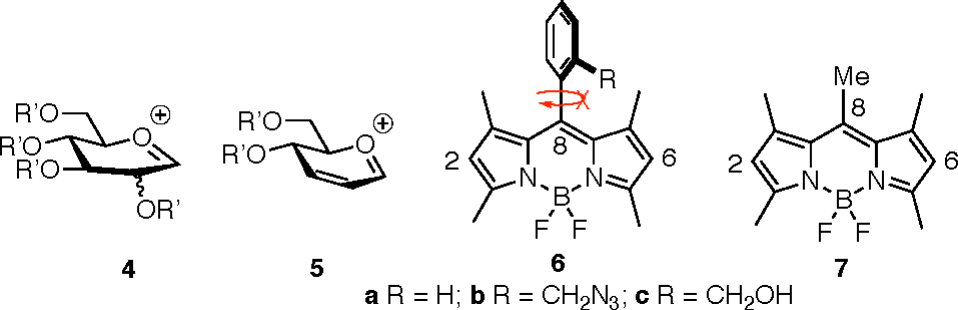
Glycosyl cation (**4**), allylic glycosyl cation (**5**), and 8-(*meso*)-substituted 1,3,5,7-tetramethyl BODIPYs **6a–c** and **7**.

Ferrier-type glycosylations involve the treatment of Δ^1,2^-unsaturated monosaccharides, 1,5-anhydrohex-1-enitols, commonly termed glycals, e.g., **8** (Scheme 1), with a Lewis acid to generate reactive electrophilic species **5**.^17,18^ Accordingly, we tested the reaction of *meso*-phenyl BODIPY **6a** with commercially available tri-*O*-acetyl-d-glucal (3,4,6-tri-*O-*acetyl-1,5-anhydro-2-deoxy-d-arabino-hex-1-enitol) **8** in the presence of three different Lewis acids, BF_3_·OEt_2_, InCl_3_, and Yb(OTf)_3_. The best results were observed when **8** (3.0 equiv) and BODIPY **6a** were treated with BF_3_·OEt_2_ (0.15 equiv) at −20 °C in dichloromethane. Under these conditions, bis-β-BODIPY-*C*-glycoside **9** was obtained as the sole isomer in 68% yield (Scheme 1a). Remarkably, the incorporation of the two glycosyl units at C2 and C6 of the BODIPY core in **9** had taken place in a completely regioselective (C1′ rather than C3′) and stereoselective manner (vide infra)^19,20^ with regard to the carbohydrate moiety (Scheme 1a). Likewise, glycosylation of 8-*o*-azidomethyl phenyl BODIPY **6b** with d-glucal **8** (4.0 equiv) provided compound **11**, again as a single regio- and stereoisomer, in 70% yield (Scheme 1b). Next, the reaction of 8-*o*-hydroxymethyl phenyl BODIPY **6c**, containing an additional hydroxyl site for glycosylation, with glycal **8** (5.0 equiv) provided tris-glycosyl BODIPY **12** as a single α,β,β stereoisomer (the *O*-glycosylation at the *meso o*-hydroxymethyl substituent was ascribed as α, according to well-established literature precedents on the Ferrier glycosylation of alcohols)^17^ in 72% yield (Scheme 1c). Conversely, the attempted glycosylation of pentamethyl BODIPY **7**, under similar reaction conditions, led only to extensive decomposition of the fluorophore.

**Scheme 1:**
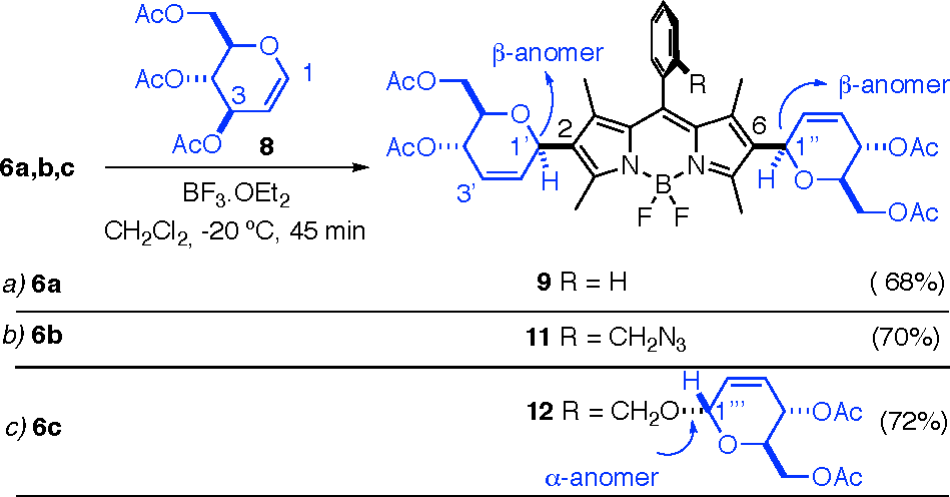
Ferrier-Type Glycosylation of BODIPYs **6a–c** with Tri-*O*-acetyl-d-glucal **8**

The β configuration at the anomeric center (C1′ or C1″) on each hex-2-eno-pyranoside moieties in compound **9** was rigorously established by hydrogenation to corresponding saturated derivative **10** (Figure 3), whose ^1^H NMR analysis allowed us to assess the axial orientation of the carbohydrate H1′ and H1″ protons.^21^ Saponification of the acyl groups in BODIPY-saccharides **9**, **11**, and **12** was performed by treatment with Et_3_N/MeOH (1:4) and led to water-soluble tetraols **13a** and **13b** and hexaol **14**, respectively (Figure 3).

**Figure 3:**
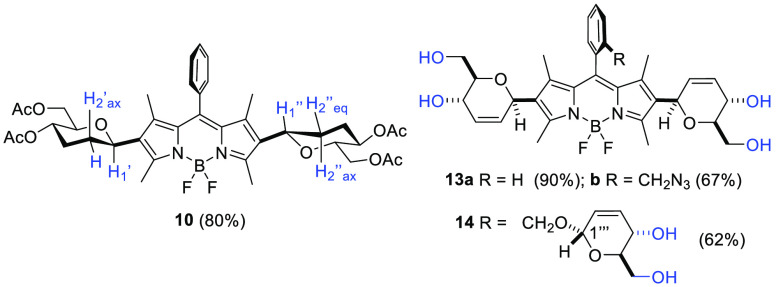
Glyco-BODIPYs **10**, **13a**, **13b**, and **14**.

Azidomethyl BODIPY **6b** was selected in this study because the ensuing *C*-glycosyl BODIPYs **11** and **13b** possess an additional site (N_3_) for conjugation.^11c,15b,22^ One example of the versatility of these compounds was provided by the one-pot dimerization of **11** and **13b**, leading to bis-urea derivatives **15** and **16**, respectively (Figure 4).^22^

**Figure 4:**
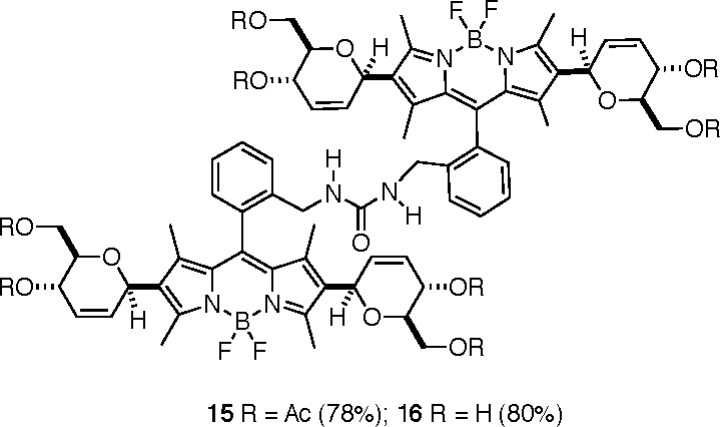
Bis-BODIPY ureas **15** and **16**, obtained by dimerization of **11** and **13b**, respectively [Et_3_NHCO_3_ (TEAB) (4.0 equiv), PPh_3_ (1.5 equiv)].

The photophysical behavior of the new glyco-BODIPYs was evaluated under both soft and laser irradiation. The small-molecule BODIPY-*C*-glycosides (acetylated **9**, **11**, and **12** and saponified **13a**, **13b**, and **14**) displayed strong absorption and fluorescence bands centered around 510 and 520 nm, respectively, showing the low negative solvatochromism distinctive of these fluorophores (Figure S1).^15b^ The conformationally restricted molecular geometry of the new glyco-dyes led to an efficient fluorescence emission with quantum yields ranging from 50% to 90% and monoexponential lifetimes ranging from 3 to 5 ns (Figure 5 and Table 1). The theoretical simulation of the optimized molecular geometries of the glyco-BODIPYs confirmed that the persubstitution of the dipyrrin system exerted the desired sterical hindrance at the 2,6-saccharides and, especially, at the 8-aryl moiety, placing all of these rings orthogonal to the chromophoric core (Figure S2). In this way, nonradiative deactivation funnels related to the free motion of the substituents were at least partially hindered, because further structural constraints still ameliorated the fluorescence response (Table 1). In fact, the 8-aryl moiety in **9** and **13a**, despite the 1,7-methylation of the dipyrrin, could retain some rotational freedom around its perpendicular disposition, decreasing the fluorescence efficiency to 50%. Further sterical hindrance asserted by *ortho* substitution at this 8-aryl moiety completely locked its slight rotational motion, fixing its structural disposition.^23^ This structural arrangement entailed a more efficient fluorescence response (≤90%) owing to a decrease in the nonradiative rate constant (Figure 5 and Table S1). Although overall the photophysical signatures of the acetylated and hydroxyl-free glyco-BODIPYs are quite similar (Table 1 and Table S1), decreases in both the absorption and the fluorescence probability are found upon deprotection of the carbohydrate moieties. The absorption spectral profile of the unprotected glyco-BODIPYs becomes slightly flattened and broadened [full width at half-maximum (fwhm) increase around 100 cm^–1^], leading to a decrease in the molar absorption coefficient that runs simulatenously with a less pronounced decrease in the corresponding oscillator strength [calculated from the area under the absorption band (see Table 1 and Table S1)].

**Table 1:** Photophysical Properties of Glyco-BODIPYs with Protected (**9**, **11**, **12**, and **15**, in ethyl acetate) and Unprotected (**13a**, **13b**, **14**, and **16**, in water) Carbohydrate Moieties (dye concentration of 2 μM)^a^

	λ_ab_ (nm)	ε_max_ (*f*) (×10^4^ M^–1^ cm^–1^)	λ_fl_ (nm)	ϕ	τ (ns)
**9**	509.0	10.4 (0.57)	521.0	0.66	3.68
**11**	512.5	8.8 (0.48)	525.0	0.91	4.93
**12**	511.5	10.9 (0.58)	522.5	0.88	5.06
**15**	510.0	14.9 (0.71)	523.5	0.59	4.72^b^
**13a**	504.5	3.5 (0.30)	517.0	0.47	3.42
**13b**	509.0	5.6 (0.40)	521.5	0.67	5.14
**14**	508.0	5.9 (0.40)	521.0	0.77	5.26
**16**	507.5	8.0 (0.50)	522.0	0.08	3.81^b^

a Full photophysical data for the saponified compounds are listed in Table S1 for the single BODIPYs and Table S3 for the bis-BODIPYs. Absorption (λ_ab_) and fluorescence (λ_fl_) wavelengths, molar absorption coefficients at the maximum (ε_max_), oscillator strengths (*f*), fluorescence quantum yields (ϕ), and lifetimes (τ) are given. Estimate errors: ±0.5 nm for wavelengths and 5% for the rest of the parameters.

b Amplitude average lifetime of the resulting biexponential fit of the decay curves (Table S3).

**Figure 5:**
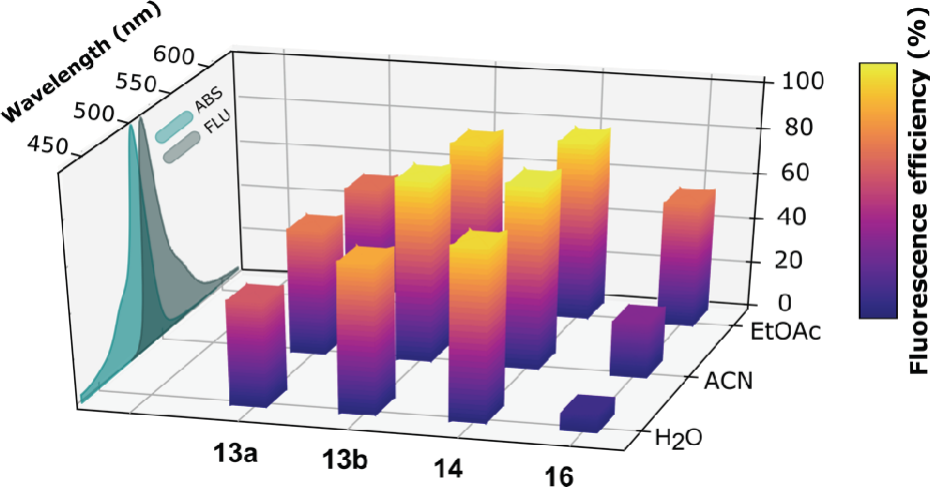
Variation of the fluorescence efficiency with the solvent (EtOAc, CH_3_CN, and H_2_O) for BODIPYs **13a**, **13b**, and **14** and bis-BODIPY **16**, all grafted to unprotected sugar units. Representative absorption and fluorescence spectra are also included.

BODIPYs grafted to unprotected sugar units (**13a**, **13b**, and **14**) were completely soluble in water in the millimolar range at room temperature (Figure S3) and retained a noticeable fluorescence efficiency [i.e., ≤77% for **14** (Figure 5 and Table 1)]. Increasing the dye concentration in water altered neither the absorption nor the fluorescence profiles, highlighting the absence of intermolecular aggregation, the most effective deactivation pathway of BODIPY in water, which is unambiguously tracked through the drastic changes induced in the spectral bands.^9c^ Therefore, these glyco-BODIPYs are not prone to aggregate even after reaching their solubility limit in water (Figure S3). The highly constrained geometrical molecular arrangement of the new glycosylated BODIPYs entailed an enhanced water solubility while hindering any intermolecular exciton coupling of these inherently hydrophobic chromophores. Thus, it appears that the conformational rigidity asserted by the direct linkage of the bulky saccharides at positions C2 and C6 and the orthogonal disposition of the 8-aryl moiety with respect to the main plane of the chromophoric framework hampered the stacking of the boradiazaindacene units in concentrated water solutions. This phenomenon was even reinforced when a third hydrophilic, bulky, sugar unit was incorporated at one of the apical positions of the BODIPY chromophore, as in **14**, thus shielding one of the faces perpendicular to the BODIPY core.^9b^ Therefore, *C*-glycosylation of the BODIPY skeleton might then be visualized as a concise and suitable strategy for fine-tuning its water solubility while keeping a notable fluorescence response even in high-optical density media.

Understanding laser-induced photophysical behavior is key in the engineering of photonic materials for advanced applications such as high-resolution microscopy techniques involving laser radiation as an excitation source. Therefore, the lasing properties of the new glycosylated derivatives were studied, according to their absorption properties, under pumping radiation at both 355 and 532 nm. Laser emission, centered in the 556–568 nm spectral region, was recorded from the new dyes with efficiencies of ≤22% (see Table S2). A further important parameter is the dye photostability over long operation times. Long-lasting efficient emitters under lasing irradiation are sought to reach the nominal resolution of the most advanced optical microscopy. The photostability of the new dyes was analyzed by the decay of its laser-induced fluorescence (LIF) intensity in an ethyl acetate solution upon severe laser pumping (see the Supporting Information for experimental details). All of the glyco-BODIPYs studied (acylated and saponified derivatives) displayed a high photostability because their LIF emission decreased by merely 10% of its initial value after 70000 pump pulses at a repetition rate of 10 Hz.

The spectral bands of the urea-bridged bis-BODIPYs featuring C2,C6 carbohydrate units (**15** and **16**) showed profiles similar to those described above for the small-molecule saccharidic BODIPYs, but with higher molar absorption coefficients, owing to the additive contribution of both chromophoric subunits in the dimer (Figure S4). Unprotected bis-BODIPY **16** showed a limited water solubility, being fully dissolved just in the micromolar range. The appended carbohydrates were likely not able to efficiently decrease the hydrophobic nature of the dimeric urea. The fluorescence response of **16** is lower than that of its corresponding monomeric precursor, especially in polar media (Figure 5 and Table S3). Similar results were previously reported for nonglycosylated urea-bridged bis-BODIPYs.^22,24^ As mentioned above, each chromophoric fragment retains its molecular identity after covalent assembly, thus showing isoenergetic excited states and electronic transitions that enable energy and electron transfer processes. The migration of energy between bridged BODIPY units could quench the fluorescence through the dissipation of excitation energy as heat, but it solely cannot explain the marked solvent-sensitive fluorescence response of **16**. Such polarity-triggered fluorescence quenching was ascribed to an additional nonradiative pathway such as photoinduced electron transfer (PET) between the pair of identical BODIPYs electronically decoupled in the ground state.^22−25^ This electron transfer mechanism with no electrostatic driving force is enhanced by the solvent polarity, thus decreasing the fluorescence efficiency in polar media (Figure 5 and Table S3).

Even though this PET process was detrimental to the fluorescence response, it paved the way to new photoinduced pathways. In fact, the ability of electron transfer to mediate in the triplet state population is actively applied to develop heavy atom-free singlet oxygen photosensitizers for photodynamic therapy (PDT).^26^ Against this background, and bearing in mind the aforementioned ongoing PET in the glycosylated bis-BODIPY,^22^ we analyzed its suitability to generate singlet oxygen in chloroform. Bis-BODIPY **16** yielded a singlet oxygen generation efficiency of 12%, while retaining a fluorescence response of 58% in this solvent (Table S3). Therefore, this bis-glyco-BODIPY could be envisaged as an effective theranostic agent allowing simultaneously imaging (fluorescence) and phototreatment (singlet oxygen generation). Note that the rest of the herein reported monomeric glycoprobes (**13** and **14**) did not display such emission, supporting the key role on the PET in mediating the population of the triplet manifold and overall in the fluorescence signatures of bis-BODIPYs.

In summary, we have reported a concise method for the incorporation of at least two sugar units at C2 and C6 into the BODIPY core in 8-aryl 1,3,5,7-tetramethyl BODIPYs by direct Ferrier-type *C*-glycosylation of the boradiaza-*s*-indacene core. The presence of an aryl group at C8 appeared to be necessary for the reaction to succeed, probably by stabilizing positively charged reactive species because a related *meso*-methyl BODIPY underwent extensive decomposition. The constrained geometry of these *C*-glycosyl BODIPYs avoids aggregation in highly concentrated aqueous media, thus showing improved water solubility while retaining a bright and stable emission. In addition, the ongoing PET in water-soluble glycosylated bis-BODIPYs enables singlet oxygen generation while retaining a fluorescence response being suited for theranostic purposes.

## Experimental Section

### General Information

All solvents and reagents were obtained commercially and used as received unless stated otherwise. Residual water was removed from starting compounds by repeated co-evaporation with toluene. All moisture-sensitive reactions were performed in dry flasks fitted with glass stoppers or rubber septa under a positive pressure of argon. Anhydrous MgSO_4_ or Na_2_SO_4_ was used to dry organic solutions during workup. Evaporation of the solvents was performed under reduced pressure using a rotary evaporator. Flash column chromatography was performed using 230–400 mesh silica gel. Thin-layer chromatography was conducted on Kieselgel 60 F254. Spots were observed first under UV irradiation (254 nm) and then by charring with a solution of 20% aqueous H_2_SO_4_ (200 mL) in AcOH (800 mL). All melting points were determined with a Stuart SMP-20 apparatus. Optical rotations were measured on a Jasco P2000 polarimeter with [α]_D_^25^ values reported in degrees with concentrations expressed in grams per 100 mL. ^1^H and ^13^C NMR spectra were recorded in CDCl_3_ or CD_3_OD at 300, 400, or 500 MHz and 75, 101, or 126 MHz, respectively. Chemical shifts are expressed in parts per million (δ) downfield from tetramethylsilane and are referenced to residual protium in the NMR solvent (CHCl_3_, δ 7.25; CH_3_OH, δ 4.87). Coupling constants (*J*) are given in hertz. All presented ^13^C NMR spectra are proton-decoupled. Mass spectra were recorded by direct injection with an Accurate Mass Q-TOF LC/MS spectrometer equipped with an electrospray ion source in positive mode.

### General Method for the BF_3_·OEt_2_-Catalyzed Ferrier Reaction of BODIPYs with Acetylated Glucal **8**

To a stirred solution of tri-*O*-acetyl-d-glucal **8** (3–5 equiv) and the appropriate BODIPY (1 equiv) in anhydrous CH_2_Cl_2_ (20 mL/mmol) was added 4 Å molecular sieves. The mixture was stirred at room temperature (rt) for ∼15 min under an Ar atmosphere and then cooled to −20 °C. BF_3_·OEt_2_ (0.15 equiv) was then added. The mixture was stirred under these conditions for 45–90 min, poured into 5 mL of a saturated aqueous NaHCO_3_ solution, and partitioned twice with 10 mL of CH_2_Cl_2_. The combined organic layers were washed once with brine, dried over MgSO_4_, and filtered, and the solvent was removed in vacuo. The crude material was purified through silica gel column chromatography.

#### 2,6-Bis(4,6-di-*O*-acetyl-2,3-dideoxy-β-d-erythro-hex-2-enopyranosyl)-8-phenyl-1,3,5,7-tetramethyl-4,4-difluoro-4-bora-3a,4a-diaza-*s*-indacene (**9**)

BODIPY **6a** (80 mg, 0.25 mmol) was reacted with tri-*O*-acetyl-d-glucal **8** (204 mg, 0.75 mmol) and BF_3_·OEt_2_ (4.6 μL, 0.04 mmol) following the general procedure (−20 °C, 45 min). The residue was purified by flash silica gel chromatography (9:1 hexane/ethyl acetate) to give derivative **9** (127 mg, 68%): red solid; mp 82–83 °C; [α]_D_^21^ = +98.1 (*c* 0.025, CHCl_3_); ^1^H NMR (300 MHz, CDCl_3_) δ 7.49–7.46 (m, 3H), 7.23–7.20 (m, 2H), 5.83–5.73 (m, 4H), 5.36–5.32 (m, 2H), 5.20–5.18 (m, 2H), 4.22 (dd, *J* = 12.1, 2.5 Hz, 2H), 4.13 (dd, *J* = 12.1, 5.3 Hz, 2H), 3.85 (ddd, *J* = 9.1, 5.3, 2.5 Hz, 2H), 2.57 (s, 6H), 2.08 (s, 6H), 2.04 (s, 6H), 1.34 (s, 6H); ^13^C{^1^H} NMR (75 MHz, CDCl_3_) δ 171.0, 170.4, 155.3, 142.6, 141.3, 135.1, 133.8, 131.6, 131.0, 129.4, 129.3, 129.0, 128.6, 128.0, 127.8, 125.2, 75.0, 69.9, 65.1, 63.5, 21.1, 20.8, 13. 4, 12.2; HRMS (ESI) *m*/*z* calcd for [M + H]^+^ C_39_H_44_BF_2_N_2_O_10_ 749.3058, found 749.3051; HRMS (ESI) *m*/*z* calcd for [M + NH_4_]^+^ C_39_H_47_BF_2_N_3_O_10_ 766.3313, found 766.3324.

#### 2,6-Bis(4,6-di-*O*-acetyl-2,3-dideoxy-β-d-erythro-hex-2-enopyranosyl)-8-(2-azidomethyl)-phenyl-1,3,5,7-tetramethyl-4,4-difluoro-4-bora-3a,4a-diaza-*s*-indacene (**11**)

BODIPY **6b** (130 mg, 0.34 mmol) was reacted with tri-*O*-acetyl-d-glucal **8** (280 mg, 1.03 mmol) and BF_3_·OEt_2_ (6 μL, 0.05 mmol) following the general procedure (−20 °C, 45 min). The residue was purified by flash silica gel chromatography (9:1 toluene/ethyl acetate) to give derivative **11** (191 mg, 70%): red solid; mp 68–69 °C; [α]_D_^21^ = −60.5 (*c* 0.025, CHCl_3_); ^1^H NMR (500 MHz, CDCl_3_) δ 7.58–7.53 (m, 2H), 7.45 (td, *J* = 7.5, 1.7 Hz, 1H), 7.19 (d, *J* = 7.5 Hz, 1H), 5.83–5.75 (m, 4H), 5.36–5.34 (m, 2H), 5.20–5.19 (m, 2H), 4.32 (bs, 2H), 4.24 (dd, *J* = 12.2, 2.3 Hz, 2H), 4.16–4.11 (m, 2H), 3.87–3.84 (m, 2H), 2.58 (s, 6H), 2.09 (s, 6H), 2.06 (s, 6H), 1.323 (s, 3H), 1.317 (s, 3H); ^13^C{^1^H} (126 MHz, CDCl_3_) δ 171.1, 170.4, 156.3, 156.1, 140.9, 140.7, 139.8, 134.0, 133.7, 131.50, 131.48, 130.2, 129.2, 129.0, 128.7, 125.42, 125.37, 75.12, 75.07, 69.98, 69.88, 65.11, 65.08, 63.5, 52.1, 21.2, 21.0, 13.6, 13.5, 11.78, 11.75; HRMS (ESI) *m*/*z* calcd for [M + H]^+^ C_40_H_45_BF_2_N_5_O_10_ 804.3229, found 804.3249.

#### 2,6-Bis(4,6-di-*O*-acetyl-2,3-dideoxy-β-d-erythro-hex-2-enopyranosyl)-8-[(4,6-di-*O*-acetyl-2,3-dideoxy-α-d-erythro-hex-2-enopyranosyl)-2-methylphenyl]-1,3,5,7-tetramethyl-4,4-difluoro-4-bora-3a,4a-diaza-*s*-indacene (**12**)

BODIPY **6c** (50 mg, 0.14 mmol) was reacted with tri-*O*-acetyl-d-glucal **8** (193 mg, 0.71 mmol) and BF_3_·OEt_2_ (3 μL, 0.02 mmol) following the general procedure (−20 °C, 90 min). The residue was purified by flash silica gel chromatography (7:3 toluene/ethyl acetate) to give derivative **12** (101 mg, 72%): red solid; mp 88–89 °C; [α]_D_^21^ = +96.8 (*c* 0.02, CHCl_3_); ^1^H NMR (400 MHz, CDCl_3_) δ 7.56 (dd, *J* = 7.2, 1.5 Hz, 1H), 7.50 (dt, *J* = 7.5, 1.0 Hz, 1H), 7.44 (dt, *J* = 7.5, 1.0 Hz, 1H), 7.17 (dd, *J* = 7.2, 1.0 Hz, 1H), 5.83–5.59 (m, 5H), 5.34–4.97 (m, 7H), 4.69 (d, *J* = 11.6 Hz, 1H), 4.36 (d, *J* = 11.6 Hz, 1H), 4.25–3.83 (m, 9H), 2.58 (s, 6H), 2.09 (s, 3H), 2.085 (s, 3H), 2.078 (s, 3H), 2.054 (s, 3H), 2.050 (s, 3H), 2.01 (s, 3H), 1.33 (s, 3H), 1.32 (s, 3H); ^13^C{^1^H} (101 MHz, CDCl_3_) δ 171.1, 170.8, 170.5, 170.3, 156.0, 155.5, 141.2, 140.6, 135.3, 134.3, 131.6, 130.7, 130.5, 129.9, 129.8, 129.7, 129.2, 128.7, 128.5, 128.4, 128.0, 127.9, 127.3, 125.4, 125.3, 94.7, 75.0, 70.0, 69.8, 68.5, 67.2, 65.1, 63.5, 62.6, 21.2, 21.14, 21.10, 20.95, 20.89, 20.85, 13.7, 13.4, 11.9, 11.7; HRMS (ESI) *m*/*z* calcd for [M + NH_4_]^+^ C_50_H_61_BF_2_N_3_O_16_ 1008.4116, found 1008.4130.

### Hydrogenation Reaction of **9**

*2,6-Bis(4,6-di-O-acetyl-2,3-dideoxy-β-*d-erythro-hex-2-pyranosyl)-8-phenyl-1,3,5,7-tetramethyl-4,4-difluoro-4-bora-3a,4a-diaza-s-indacene (**10**). A solution of compound **9** (40 mg, 0.05 mmol) in a MeOH/CH_2_Cl_2_ mixture [3 mL, 1:1 (v/v)] was hydrogenated in a Parr hydrogenator with 10% Pd:C [10% (w/w)] at 25 psi. After reaction for 16 h, the catalyst was filtered off, the filtrate evaporated under reduced pressure, and the residue purified by flash chromatography (9:1 hexane/ethyl acetate) to give **10** (31 mg, 80%): red oil; [α]_D_^21^ = +212.3 (*c* 0.13, CHCl_3_); ^1^H NMR (300 MHz, CDCl_3_) δ 7.49–7.47 (m, 2H), 7.26–7.23 (m, 3H), 4.75 (dt, *J* = 10.0, 4.7 Hz, 2H), 4.39 (dd, *J*_1,2_ = 11.6, 2.5 Hz, 2H), 4.18–4.16 (m, 4H), 3.59 (dt, *J* = 10.0, 3.4 Hz, 2H), 2.63 (s, 6H), 2.31–2.24 (m, 2H), 2.05 (s, 6H), 2.04 (s, 6H), 2.00–1.82 (m, 2H), 1.74–1.49 (m, 4H) 1.35 (s, 6H); ^13^C NMR (101 MHz, CDCl_3_) δ 171.4, 170.6, 154.6, 142.4, 139.9, 135.6, 131.4, 130.2, 129.7, 129.5, 128.5, 78.2, 73.8, 68.1, 63.6, 30.9, 30.0, 21.6, 21.3, 14.0, 12.6; HRMS (ESI) *m*/*z* calcd for [M + H]^+^ C_39_H_48_BF_2_N_2_O_10_ 753.3371, found 753.3365.

### General Method for the Methanolysis of Acetate Esters

A solution of the corresponding acetate (0.1 mmol) in MeOH (2 mL) was treated with Et_3_N (0.5 mL). The mixture was warmed at 60 °C (heat-on blocks) and stirred at that temperature overnight. The solution was concentrated *in vacuo*, and the residue was then purified by flash column chromatography (eluent, dichloromethane/methanol mixtures).

#### 2,6-Bis(2,3-dideoxy-β-d-erythro-hex-2-enopyranosyl)-8-phenyl-1,3,5,7-tetramethyl-4,4-difluoro-4-bora-3a,4a-diaza-*s*-indacene (**13a**)

BODIPY **9** (45 mg, 0.06 mmol) was deacylated according to the general procedure (60 °C, heat-on block, overnight). The residue was purified by flash silica gel chromatography (95:5 dichloromethane/methanol) to give derivative **13a** (31 mg, 90%): red solid; mp 160–161 °C; [α]_D_^21^ = +473.1 (*c* 0.03, CH_3_OH); ^1^H NMR (500 MHz, CD_3_OD) δ 7.58–7.55 (m, 3H), 7.33–7.30 (m, 2H), 5.85 (dt, *J* = 10.5, 2.0 Hz, 2H), 5.68 (dt, *J* = 10.5, 2 Hz, 2H), 5.21–5.20 (m, 2H), 4.09–4.06 (m, 2H), 3.87 (dd, *J* = 12.0, 2.0 Hz, 2H), 3.65 (dd, *J* = 12.0, 6.5 Hz, 2H), 3.46 (ddd, *J* = 12.0, 6.5, 2.5 Hz, 2H), 2.53 (s, 6H), 1.39 (s, 6H); ^13^C{^1^H} (126 MHz, CD_3_OD) δ 155.2, 142.5, 140.7, 135.0, 130.5, 129.40, 129.37, 129.1, 128.98, 128.96, 127.95, 80.8, 69.3, 62.7, 61.9, 12.2, 10.9; HRMS (ESI) *m*/*z* calcd for [M + H]^+^ C_31_H_36_BF_2_N_2_O_6_ 581.2634, found 581.2621.

#### 2,6-Bis(2,3-dideoxy-β-d-erythro-hex-2-enopyranosyl)-8-(2-azidomethyl)-phenyl-1,3,5,7-tetramethyl-4,4-difluoro-4-bora-3a,4a-diaza-*s*-indacene (**13b**)

BODIPY **11** (59 mg, 0.07 mmol) was deacylated according to the general procedure (60 °C, heat-on block, overnight). The residue was purified by flash silica gel chromatography (95:5 dichloromethane/methanol) to give derivative **13b** (31 mg, 67%): mp 135–136 °C; [α]_D_^21^ = +1090.2 (*c* 0.025, CH_3_OH); ^1^H NMR (400 MHz, CD_3_OD) δ 7.60–7.50 (m, 3H), 7.24–7.27 (m, 1H), 5.82 (d, *J* = 10.2 Hz, 2H), 5.66 (dt, *J* = 10.2, 2.0 Hz, 2H), 5.17 (bs, 2H), 4.28–4.30 (m, 2H), 4.06–4.02 (m, 2H), 3.86–3.82 (m, 2H), 3.65–3.60 (m, 2H), 3.45–3.42 (m, 2H), 2.53 (s, 6H), 1.36 (s, 6H); ^13^C{^1^H} (101 MHz, CD_3_OD) δ 155.9, 155.8, 140.64, 140.59, 139.8, 134.2, 134.1, 130.2, 129.8, 129.60, 129.56, 129.37, 129.33, 129.28, 129.2, 128.8, 80.93, 80.90, 69.35, 69.33, 62.81, 62.78, 61.9, 51.8, 12.4, 10.7, 10.6; HRMS (ESI) *m*/*z* calcd for [M + H]^+^ C_32_H_37_BF_2_N_5_O_6_ 636.2805, found 636.2776; HRMS (ESI) *m*/*z* calcd for [M + NH_4_]^+^ C_32_H_40_BF_2_N_6_O_6_ 653.3070, found 653.3042.

#### 2,6-Bis(2,3-dideoxy-β-d-erythro-hex-2-enopyranosyl)-8-[(2,3-dideoxy-α-d-erythro-hex-2-enopyranosyl)-2-methyl-phenyl]-1,3,5,7-tetramethyl-4,4-difluoro-4-bora-3a,4a-diaza-*s*-indacene (**14**)

BODIPY **12** (42 mg, 0.04 mmol) was deacylated according to the general procedure (60 °C, heat-on block, overnight). The residue was purified by flash silica gel chromatography (9:1 dichloromethane/methanol) to give derivative **14** (18 mg, 62%): red solid; mp 148–150 °C; [α]_D_^21^ = +354.6 (*c* 0.04, CH_3_OH); ^1^H NMR (400 MHz, CD_3_OD) δ 7.63 (d, *J* = 7.5 Hz, 1H), 7.55–7.46 (m, 2H), 7.20 (d, *J* = 7.5 Hz, 1H), 5.92–5.62 (m, 4H), 5.53 (d, *J* = 10.3 Hz, 1H), 5.17 (s, 2H), 4.72–4.60 (m, 4H), 4.29 (d, *J* = 11.2 Hz, 1H), 4.06–4.01 (m, 3H), 3.84 (d, *J* = 11.5 Hz, 2H), 3.66–3.56 (m, 6H), 2.52 (s, 6H), 1.35 (s, 3H), 1.33 (s, 3H); ^13^C{^1^H} (101 MHz, CD_3_OD) δ 156.7, 156.6, 142.7, 142.2, 137.0, 135.7, 134.9, 131.6, 131.0, 130.9, 130.8, 130.6, 130.3, 130.2, 129.4, 126.8, 95.6, 82.3, 82.2, 73.5, 70.70, 70.67, 68.8, 64.1, 63.7, 63.3, 63.2, 62.2, 13.6, 12.03, 11.99; HRMS (ESI) *m*/*z* calcd for [M + NH_4_]^+^ C_38_H_49_BF_2_N_3_O_10_ 756.3480, found 756.3479.

### General Method for the Ureation Reaction

The appropriate azidomethyl-BODIPY (1 mmol) was added to a mixture of 1 M triethylammonium hydrogen carbonate buffer (TEAB) (2.6 mL) and 1,4-dioxane (6 mL) at room temperature. Triphenylphosphine (1.3 equiv) was added, and the reaction was monitored by TLC. After disappearance of the starting material, the solvent was evaporated *in vacuo* to dryness. The obtained BODIPY dimers were purified by flash chromatography on silica gel.

### Compound **15**

Azidomethyl-BODIPY **11** (40 mg, 0.05 mmol) was reacted with PPh_3_ (20 mg, 0.075 mmol) and TEAB (200 μL, 1 M solution, 0.2 mmol) following the general procedure (rt, overnight). The residue was purified by flash silica gel chromatography (7:3 hexane/ethyl acetate) to give derivative **15** (31 mg, 80%): red solid; mp 91–92 °C; [α]_D_^21^ = +563.0 (*c* 0.02, CHCl_3_); ^1^H NMR (300 MHz, CDCl_3_) δ 7.47 (d, *J* = 8.0 Hz, 2H), 7.43 (t, *J* = 7.5 Hz, 2H), 7.33 (t, *J* = 7.5 Hz, 2H), 7.08 (d, *J* = 7.8 Hz, 2H), 5.79–5.68 (m, 8H), 5.33–5.16 (m, 10H), 4.28–3.80 (m, 16H),, 2.52 (s, 6H), 2.50 (s, 6H), 2.07 (s, 12H), 2.02 (s, 6H), 2.00 (s, 6H), 1.31 (s, 6H), 1.26 (s, 3H); ^13^C{^1^H} NMR (126 MHz, CDCl_3_) δ 171.2, 171.0, 170.4, 158.0, 155.5, 155.4, 141.3, 141.15, 141.11, 137.5, 137.4, 132.9, 132.9, 132.1, 132.0, 131.55, 131.50, 130.48, 130.42, 129.9, 128.7, 128.6, 128.5, 128.15, 128.10, 128.08, 128.0, 125.12, 125.08, 75.03, 74.97, 69.9, 69.8, 65.1, 65.0, 63.4, 63.3, 41.7, 21.1, 20.94, 20.89, 20.76, 13.3, 13.2, 11.71, 11.69; HRMS (ESI) *m*/*z* calcd for [M + H]^+^ C_81_H_91_B_2_F_4_N_6_O_21_ 1581.6378, found 1581.6376; HRMS (ESI) *m*/*z* calcd for [M + NH_4_]^+^ C_81_H_94_B_2_F_4_N_7_O_21_ 1598.6643, found 1598.6628.

### Compound **16**

Azidomethyl-BODIPY **14** (26 mg, 0.04 mmol) was reacted with PPh_3_ (16 mg, 0.06 mmol) and TEAB (170 μL, 0.16 mmol) following the general procedure (rt, overnight). The residue was purified by flash silica gel chromatography (9:1 dichloromethane/methanol) to give derivative **16** (19 mg, 78%): red solid; mp >300 °C; [α]_D_^21^ +664.2 (*c* 0.03, CH_3_OH); ^1^H NMR (400 MHz, CD_3_OD) δ 7.52–7.41 (m, 6H), 7.17 (dd, *J* = 7.5, 1.4 Hz, 2H), 5.85–5.79 (m, 6H), 5.70 (dt, *J* = 10.2, 1.8 Hz, 2H), 5.21–5.18 (m, 4H), 4.25 (d, *J* = 16.2 Hz, 2H), 4.11–4.03 (m, 6H), 3.87–3.70 (m, 4H), 3.68–3.60 (m, 4H), 3.49–3.41 (m, 4H), 2.51 (s, 6H), 2.46 (s, 6H), 1.39 (s, 6H), 1.36 (s, 6H); ^13^C{^1^H} NMR (101 MHz, CD_3_OD) δ 159.8, 156.9, 156.4, 142.24, 142.16, 141.6, 139.3, 134.7, 131.6, 131.3, 130.8, 130.74, 130.70, 130.64, 130.60, 130.5, 130.2, 129.5, 129.4, 129.1, 82.2, 82.1, 70.8, 70.7, 64.1, 64.0, 63.3, 42.4, 13.7, 11.98, 11.92, 11.89; HRMS (ESI) *m*/*z* calcd for [M + H]^+^ C_65_H_75_B_2_F_4_N_6_O_13_ 1245.5529, found 1245.5552; HRMS (ESI) *m*/*z* calcd for [M + Na]^+^ C_65_H_74_B_2_F_4_N_6_NaO_13_ 1267.5348, found 1267.5388.

### Photophysical Properties

The photophysical properties were registered in diluted solutions (∼2 × 10^–6^ M) and prepared by adding the corresponding solvent (spectroscopic grade, used without furher purification or drying) to the residue from the adequate amount of a concentrated stock solution in acetone, after vacuum evaporation of this solvent. UV–vis absorption and fluorescence (after excitation at 480 nm) spectra were recorded on a Varian model CARY 4E spectrophotometer and an Edinburgh Instruments spectrofluorimeter (model FLSP 920), respectively, using quartz cuvettes with an optical path length of 1 cm. Fluorescence quantum yields (ϕ) were obtained using PM567 (laser grade from Exciton, ϕ = 0.84 in ethanol) as a reference, from corrected spectra (detector sensibility to the wavelength). The values were corrected by the refractive index of the solvent. Radiative decay curves were registered with the time-correlated single-photon counting technique as implemented in the aforementioned spectrofluorimeter. Fluorescence emission was monitored at the maximum emission wavelength (520–525 nm) after excitation (at 500 nm) by means of a Fianium pulsed laser (time resolution of picoseconds) with a tunable wavelength. The fluorescence lifetime (*t*) was obtained after the deconvolution of the instrumental response signal from the recorded decay curves by means of an iterative method. The goodness of the exponential fit was controlled by statistical parameters (χ^2^ and the analysis of the residuals). The radiative (*k*_fl_) and nonradiative (*k*_nr_) rate constants were calculated from the fluorescence quantum yield and average lifetime; *k*_fl_ = ϕ/τ, and *k*_nr_ = (1 – ϕ)/τ.

The photophysical properties at high concentrations in aqueous solutions (Milli-Q water) were recorded using cuvettes with the required optical path length (*l*) to minimize the re-absorption/re-emission phenomena at each concentration (10^–4^ M – *l* = 0.01 cm, and 2 × 10^–5^ M – *l* = 0.1 cm). The fluorescence spectra were recorded in the front-face configuration.

The photoinduced production of singlet oxygen (^1^O_2_) was determined by direct measurement of the luminescence at 1276 nm with a NIR detector integrated in the aforementioned spectrofluorometer (InGaAs detector, Hamamatsu G8605-23). The ^1^O_2_ signal was registered in the front configuration (front face), 40° and 50° to the excitation and emission beams, respectively, and leaned 30° to the plane formed by the direction of incidence and registration in cells of 1 cm. The signal was filtered by a low cutoff of 850 nm. The ^1^O_2_ generation quantum yield (ϕ^Δ^) was determined using the equation
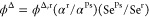
where ϕ^Δ,r^ is the quantum yield of ^1^O_2_ generation for the used reference (in our case, phenalenone). Factor α = 1 – 10^–Abs^ corrects the different amount of photons absorbed by the sample (α^Ps^) and reference (α^R^). Factor Se is the intensity of the ^1^O_2_ phosphorescence signal of the sample (Se^Ps^) and the reference (Se^r^) at 1276 nm. Phenalenone in chloroform was used as a reference for visible irradiation (420 nm), its singlet oxygen quantum yield being ϕ^Δ^ = 0.98. ^1^O_2_ quantum yields were averaged from five concentrations between 10^–6^ and 10^–5^ M in chloroform (spectroscopic grade).

### Quantum Mechanic Calculations

Ground state geometries were optimized with the b3lyp hybrid functional, within density functional theory, using the triple valence basis set with one polarization function (6-311g*). The geometries were considered as energy minima when the corresponding frequency analysis did not give any negative value. All of the calculations were conducted with Gaussian 16.

### Lasing Properties

The laser efficiency was evaluated from concentrated solutions (millimolar) of dyes in ethyl acetate contained in 1 cm optical path length rectangular quartz cells carefully sealed to avoid solvent evaporation during experiments. The liquid solutions were transversely pumped with 5 mJ, 8 ns fwhm pulses from the second (532 nm) and third (355 nm) harmonics of a Q-switched Nd:YAG laser (Lotis TII 2134) at a repetition rate of 1 Hz. The exciting pulses were line-focused onto the cell using a combination of positive and negative cylindrical lenses (*f* = 15 and −15 cm, respectively) perpendicularly arranged. The plane parallel oscillation cavity (2 cm length) consisted of a 90% reflectivity aluminum mirror acting as the back reflector, and the lateral face of the cell acting as the output coupler (4% reflectivity). The pump and output energies were detected by a GenTec power meter. The photostability of the dyes in an ethyl acetate solution was evaluated by using a pumping energy and geometry exactly equal to those of the laser experiments. We used spectroscopic quartz cuvettes with a 0.1 cm optical length to allow the minimum solution volume (40 μL) to be excited. The lateral faces were grounded, whereupon no laser oscillation was obtained. Information about photostabilitiy was obtained by monitoring the decrease in the laser-induced fluorescence (LIF) intensity after 70000 pump pulses and a repetition rate of 10 Hz to accelerate the experimental running. The fluorescence emission and laser spectra were monitored perpendicular to the exciting beam, collected by an optical fiber, imaged with a spectrometer (Acton Research Corp.), and detected with a charge-coupled device (SpectruMM:GS128B). The fluorescence emission was recorded by feeding the signal into the boxcar (Stanford Research, model 250) to be integrated before being digitized and processed by a computer. The estimated error in the energy and photostability measurements was 10%.

## Supporting Information Available

Copies of ^1^H and ^13^C NMR spectra, photophysical studies for some of the derivatives, and atom coordinates and total energies (in hartrees) in the ground state (b3lyp/6-311G*) for glyco-BODIPYs (**13a**, **13b**, **14**, and **16**) (PDF)

## Dedication

This article is dedicated to the memory of our mentor (A.M.G., J.C.L.) Prof. Bert Fraser-Reid (1934−2020).
