# Supplementary material for: A Concise Route to Water-Soluble 2,6-Disubstituted BODIPY-Carbohydrate Fluorophores by Direct Ferrier-Type C-Glycosylation
Source: J Org Chem. 2021 Jun 22;86(13):9181–8. doi: 10.1021/acs.joc.1c00413 (PMC8279486; doi:10.1021/acs.joc.1c00413)
Supplement: Supplementary file 1 — jo1c00413_si_001.pdf [file jo1c00413_si_001.pdf]

A concise route to water-soluble 2,6-disubstituted BODIPY-carbohydrate fluorophores by direct Ferrier-type *C*-glycosylation.

Ana M. Gómez,[$]* Clara Uriel,[$] Ainhoa Oliden-Sánchez,[§] Jorge Bañuelos,[§]* Inmaculada Garcia-Moreno,[¶] and J. Cristobal López,[$]*

[$] *Instituto de Química Orgánica General,* IQOG-CSIC*, Juan de la Cierva 3, 28006, Madrid, Spain.*

[§] *Departamento de Química Física. Universidad del Pais Vasco,* UPV-EHU*, Apartado 644, 48080, Bilbao, Spain.*

[¶] *Instituto de Química-Física Rocasolano, CSIC, Serrano 119, 28006, Madrid, Spain.*

Supporting Information

Table of Contents

1. **Photophysical Data**
   Table S1. Photophysical properties of single glyco-BODIPYs…………….. S2
   Table S2. Laser properties of glyco-BODIPYs…………………………….. S3
   Table S3. Photophysical properties of bis glyco-BODIPYs………………. S3
   Figure S1. Absorption and normalized fluorescence spectra………………. S4
   Figure S2. Optimized (b3lyp/6-311g*) ground state geometries…………… S4
   Figure S3. Normalized absorption and fluorescence spectra of glyco-BODIPYs………………………………………………………………………. S5
   Figure S4. Absorption and fluorescence spectra, and decay curves of bis-BODIPYs……………………………………………………………………. S5
2. **Quantum mechanics calculations.** Atom coordinates and total energy (in hartrees) in the ground state (b3lyp/6-311G*) for glyco-BODIPYs (**13a**, **13b**, **14** and **16**). **Table S4**……………………………………………………. S6-S9
3. **Copies of ^{1}H NMR and ^{13}C NMR spectra.** Figures **S5** to **S23**……….. S10-S19

1. Photophysical Data

Table S1. Photophysical properties of single glyco-BODIPYs (protected **9**, **11** and **12** in ethyl acetate and unprotected **13a**, **13b** and **14** in different solvents) in diluted solutions (dye concentration 2 μM).

	λ_{ab} (nm)	ε_{max} (*f*) (10^4 $M^{-1}\cdot cm^{-1}$)	λ_{fl} (nm)	Φ	τ (ns)	k_{fl} (10^8 s^{-1})	k_{nr} (10^8 s^{-1})
**9**							
Ethyl acetate	509.0	10.4 (0.57)	521.0	0.66	3.68	1.79	0.92
**11**							
Ethyl acetate	512.5	8.8 (0.48)	525.0	0.91	4.93	1.84	0.18
**12**							
Ethyl acetate	511.5	10.9 (0.58)	522.5	0.88	5.06	1.74	0.23
**13a**							
Ethyl acetate	510.5	6.2 (0.45)	522.0	0.53	3.66	1.45	1.28
Acetonitrile	508.0	5.4	521.0	0.56	3.63	1.54	1.21
Water	504.5	3.5 (0.30)	517.0	0.47	3.42	1.37	1.55
**13b**							
Ethyl acetate	513.5	6.3 (0.40)	524.5	0.76	4.95	1.53	0.48
Acetonitrile	512.0	6.3	524.0	0.81	5.14	1.57	0.37
Water	509.0	5.6 (0.40)	521.5	0.67	5.14	1.30	0.64
**14**							
Ethyl acetate	511.0	5.5 (0.38)	523.0	0.80	5.04	1.58	0.39
Acetonitrile	510.0	6.3	522.5	0.82	5.28	1.55	0.34
Water	508.0	5.9 (0.40)	521.0	0.77	5.26	1.46	0.43

Absorption (λ_{ab}) and fluorescence (λ_{fl}) wavelength, molar absorption at the maximum (ε_{max}), oscillator strength (*f*), fluorescence quantum yield (ϕ) and lifetime (τ), radiative (k_{fl}) and non-radiative (k_{nr}) rate constants.

Table S2. Laser properties of glyco-BODIPYs (protected **9**, **11** and **12** and unprotected **13a, 13b** and **14**) in concentrated solutions (mM) of ethyl acetate under pumping at the second (532 nm) and third (355 nm) harmonic of the Nd:YAG laser.

	λ_{la} (nm)	%Eff 532 nm	%Eff 355 nm
**9**	568	8	10
**11**	556	5	8
**12**	560	16	22
**13a**	568	0	3
**13b**	556	0	1
**14**	560	0	8

Laser wavelength (λ_{la}) and efficiency (%Eff).

Table S3. Photophysical properties of the bis glyco-BODIPY (protected **15** in ethyl acetate and unprotected **16** in different solvents) in diluted solutions (2 μM).

	λ_{ab} (nm)	ε_{max} (*f*) (10^4 $M^{-1}\cdot cm^{-1}$)	λ_{fl} (nm)	Φ	τ (ns)
**15**					
Ethyl acetate	510.0	14.9 (0.71)	523.5	0.59	2.84 (15%) / 5.05 (85%)
**16**					
Chloroform	511.5	10.0	526.0	0.58	2.50 (23%) / 5.49 (77%)
Ethyl acetate	511.0	11.9 (0.64)	525.5	0.56	2.70 (17%) / 5.19 (83%)
Ethanol	510.5	12.9	525.0	0.35	0.20 (37%) / 4.69 (63%)
DMF	513.0	12.6	528.0	0.54	4.69
Acetonitrile	510.0	12.8	524.5	0.25	2.26 (65%) / 4.28 (35%)
Water	507.5	8.0 (0.49)	522.0	0.08	1.50 (32%) / 4.90 (68%)

DMF: dimethylformamide

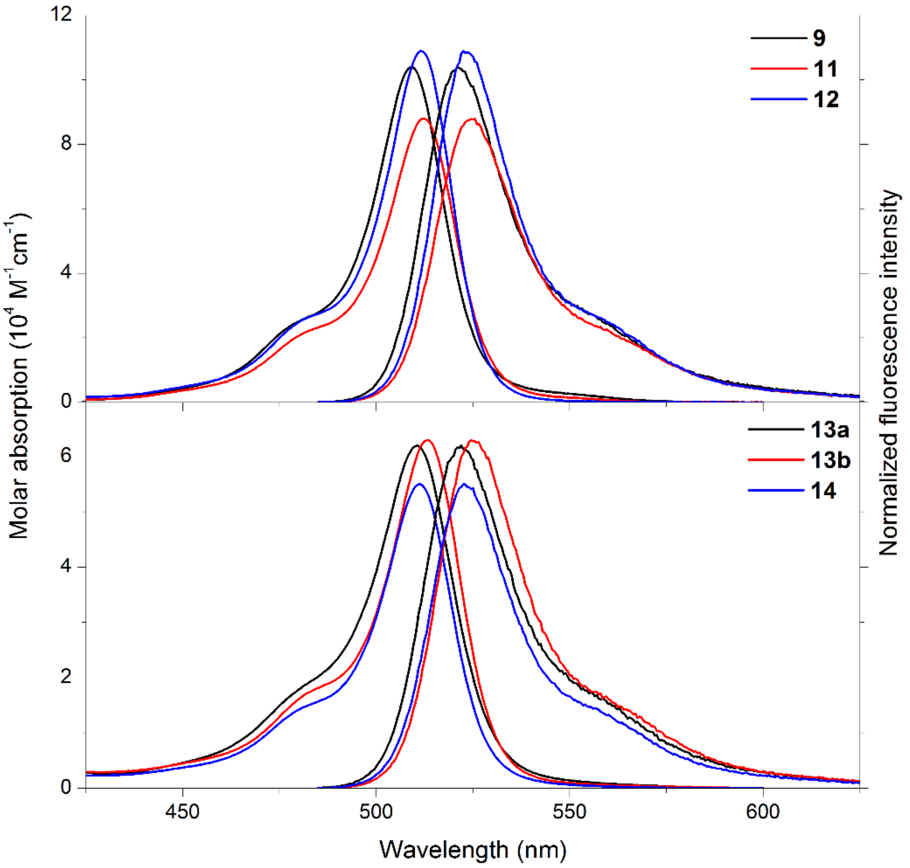

Figure S1. Absorption and normalized fluorescence spectra of the single glyco-BODIPYs bearing protected (top), and unprotected (bottom) carbohydrate units in diluted (micromolar) solutions of ethyl acetate.

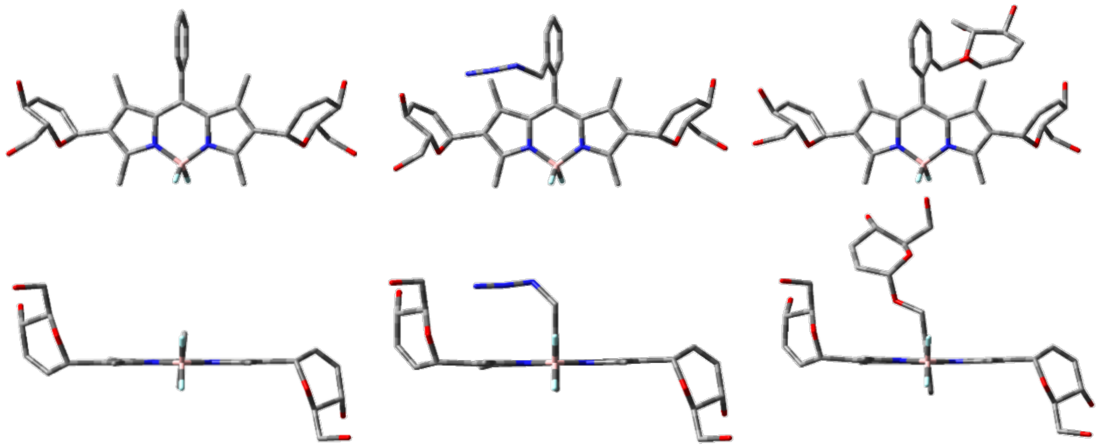

Figure S2. Optimized (b3lyp/6-311g*) ground state geometries of the unprotected glyco-BODIPYs (**13a, 13b** and **14** from left to right). For a better visualization of the disposition of the peripheral rings with respect to the dipyrrin plane the geometries are depicted in two different views; frontal (top) and from the boron atom (bottom).

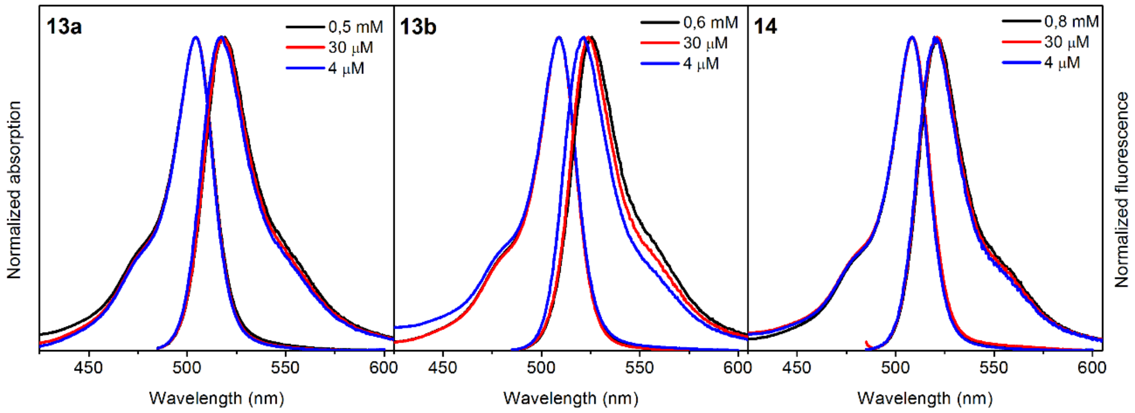

Figure S3. Normalized absorption and fluorescence spectra of unprotected glyco-BODIPYs **13a, 13b** and **14**, in water increasing the dye concentrations.

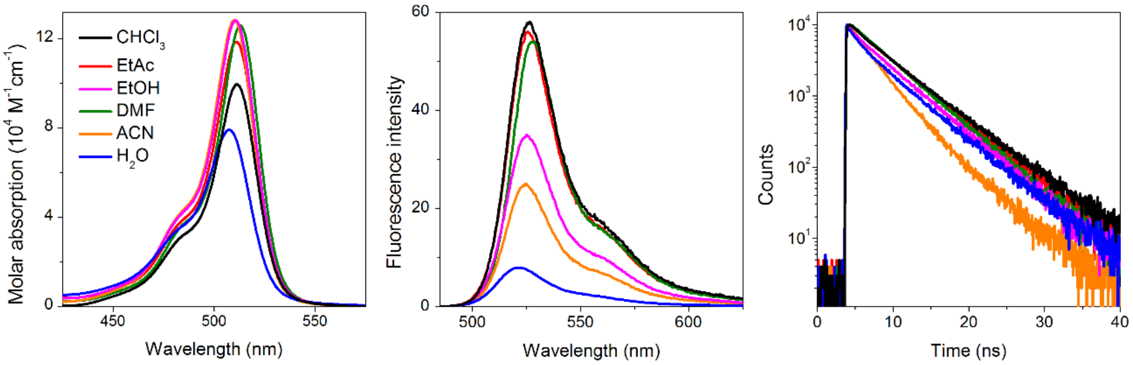

Figure S4. Absorption and fluorescence (scaled by its efficiency) spectra, and decay curves of the bis glyco-BODIPY **16** bearing carbohydrates in diluted solutions (micromolar) of different solvents.

2. Quantum mechanics calculations.

Table S4. Atom coordinates and total energy (in hartrees) in the ground state (b3lyp/6-311G*) for the BODIPYs (**13a**, **13b** and **14**) and bis-BODIPY (**16**) bearing unprotected carbohydrates.

**13a**	C	-1.18599239	0.07915737	-0.31033677	**13b**	C	-0.98514894	-0.22500481	-0.69914997
	N	-1.20493107	-1.31894297	-0.31874459		N	-1.00360433	-1.60116016	-0.45372135
	B	-0.00008963	-2.24754620	0.00046559		B	0.19340873	-2.44984797	0.06319850
	N	1.20494967	-1.31894628	0.31897500		N	1.38063418	-1.46518377	0.26825390
	C	1.18600576	0.07913757	0.31059173		C	1.34019395	-0.08271820	0.06605124
	C	0.00000958	0.76641741	0.00014067		C	0.16883947	0.52515432	-0.41715221
	C	-2.50475065	0.53535124	-0.65543934		C	-2.28559892	0.14766079	-1.18998046
	C	-3.26992586	-0.61188087	-0.89016419		C	-3.05283391	-1.02115059	-1.20546148
	C	-2.43203888	-1.73512348	-0.65674686		C	-2.21911782	-2.08062234	-0.74848048
	C	2.43208998	-1.73513005	0.65687588		C	2.59049497	-1.80096920	0.73362159
	C	3.27000627	-0.61188201	0.89019611		C	3.39396941	-0.63535026	0.86212088
	C	2.50480886	0.53534740	0.65556741		C	2.62528081	0.45011212	0.43067356
	C	0.00000458	2.26093981	0.00019367		C	0.14453263	2.00874866	-0.61676546
	C	-0.19090681	2.96896053	1.19024871		C	-0.23921306	2.88374198	0.41324481
	C	-0.19431802	4.36195475	1.18961789		C	-0.24309871	4.26119846	0.16720727
	C	-0.00000913	5.06189938	0.00033901		C	0.11973991	4.77025244	-1.07443678
	C	0.19430756	4.36207823	-1.18901102		C	0.49730732	3.90037496	-2.09574254
	C	0.19090944	2.96908351	-1.18978409		C	0.50819226	2.52998678	-1.86333781
	F	0.28243358	-3.05052668	-1.11086012		F	0.52390902	-3.42404875	-0.88331671
	F	-0.28279145	-3.04963667	1.11237720		F	-0.12833733	-3.05051747	1.28559645
	C	-2.97366930	1.95969625	-0.73531395		C	-2.70320411	1.50715496	-1.67386480
	C	-2.79781937	-3.17899916	-0.76319317		C	-2.56468824	-3.52742331	-0.62192595
	C	2.97370155	1.95969428	0.73549090		C	3.06163467	1.88433815	0.34350227
	C	2.79793065	-3.17897853	0.76348355		C	2.97293197	-3.20618669	1.06262369
	C	4.71812701	-0.81778970	1.29840959		C	4.82026855	-0.75260160	1.37115750
	C	-4.71797218	-0.81775906	-1.29864802		C	-4.50972363	-1.30039400	-1.55133669
	O	-5.50203308	-1.33161816	-0.20838933		O	-5.20304177	-1.80531888	-0.39858942
	C	-6.04727911	-0.32965131	0.64289975		C	-5.65261316	-0.79094445	0.49582346
	C	-7.07213860	0.50224833	-0.13375505		C	-6.72012846	0.08514864	-0.18022141
	C	-6.51032662	0.88789063	-1.47823844		C	-6.32035569	0.39712565	-1.59816198
	C	-5.40847200	0.33564047	-1.97758751		C	-5.29138856	-0.19012906	-2.20545909
	O	5.50196736	-1.33163921	0.20796572		O	5.67359404	-1.36588865	0.39066753
	C	6.04712861	-0.32965113	-0.64335817		C	6.25013503	-0.45272503	-0.53732968
	C	7.07218088	0.50212199	0.13318488		C	7.21225304	0.48690501	0.19585321
	C	6.51054850	0.88783124	1.47771833		C	6.56524394	1.00895097	1.45295147
	C	5.40876003	0.33559460	1.97723294		C	5.44728271	0.48810042	1.95056672
	C	-6.63046943	-1.00556334	1.86911611		C	-6.15095155	-1.45344063	1.76564050
	O	-7.78805768	-1.74685682	1.49604350		O	-7.30174151	-2.23489230	1.46838296
	C	6.63007535	-1.00551752	-1.86971899		C	6.91729747	-1.24848309	-1.64296004
	O	7.78790364	-1.74660605	-1.49695738		O	8.06705644	-1.90824068	-1.12269678
	O	-7.33821294	1.66995844	0.65985520		O	-6.95738307	1.27516859	0.57639029
	O	7.33842075	1.66987186	-0.66033252		O	7.50619597	1.56428971	-0.70773062
	H	-0.34210720	2.42423560	2.11663832		C	-0.62401549	2.37285025	1.79215458
	H	-0.34798569	4.90028216	2.11937628		N	-1.81683979	3.05176445	2.36341027
	H	-0.00001461	6.14702606	0.00039287		N	-2.90888513	2.72874450	1.90778239
	H	0.34797527	4.90050281	-2.11871343		N	-3.97617186	2.51831725	1.58152293
	H	0.34212422	2.42445843	-2.11623094		H	-0.54938187	4.93545228	0.96029131
	H	-2.65460515	2.54013240	0.13042161		H	0.10775098	5.84178022	-1.24443608
	H	-2.57446437	2.47086226	-1.61634285		H	0.78031778	4.28709437	-3.06917378
	H	-4.05774908	2.00952241	-0.78799977		H	0.79943027	1.84608084	-2.65388349
	H	-3.86674965	-3.31428495	-0.60219201		H	-2.26377125	1.72466600	-2.65284444
	H	-2.53742622	-3.57415338	-1.75104294		H	-3.78211642	1.56964280	-1.77474901
	H	-2.25289864	-3.76932952	-0.02796028		H	-2.38688595	2.30859906	-1.01017789
	H	4.05778888	2.00954997	0.78800369		H	-3.64182394	-3.65928369	-0.54654374
	H	2.65446630	2.54022852	-0.13011051		H	-2.19357073	-4.08491859	-1.48842524
	H	2.57464162	2.47073728	1.61666126		H	-2.09120387	-3.95972758	0.25919026
	H	2.25206077	-3.76964226	0.02923938		H	4.11559901	1.98537346	0.58616279
	H	3.86666477	-3.31434695	0.60123429		H	2.91276314	2.29151986	-0.65765108

H	2.53882732	-3.57363080	1.75188138
H	4.72554559	-1.64609621	2.01669708
H	-4.72527006	-1.64603045	-2.01698058
H	-5.25392099	0.35016202	0.98738110
H	-7.98634669	-0.09023882	-0.24837988
H	-7.02425139	1.68539341	-2.01069416
H	-4.98701328	0.66890171	-2.92243015
H	5.25375312	0.35023094	-0.98766542
H	7.98628896	-0.09051724	0.24774578
H	7.02455000	1.68538189	2.01001230
H	4.98743200	0.66889017	2.92212146
H	-6.87931154	-0.22653722	2.60086505
H	-5.86015892	-1.66040196	2.29523324
H	-8.09332972	-2.24025464	2.26369637
H	6.87859440	-0.22649605	-2.60158413
H	5.85974579	-1.66049019	-2.29558339
H	8.09246277	-2.24076445	-2.26440185
H	-8.22098341	1.98967049	0.44820331
H	8.22226111	1.98787900	-0.45063777

E = -1988.3021

H	2.49993410	2.52848175	1.02590679
H	2.45097611	-3.91004159	0.41665598
H	4.04705051	-3.34553163	0.94302157
H	2.70143800	-3.45100411	2.09531553
H	4.80267042	-1.49324681	2.17924338
H	-4.52373500	-2.15147229	-2.24253394
H	-4.80383428	-0.14630806	0.77524235
H	-7.67331136	-0.44757307	-0.16733335
H	-6.90940140	1.15955064	-2.10208255
H	-5.00788672	0.07913802	-3.21970018
H	5.46629213	0.16411268	-1.00144583
H	8.12712298	-0.06738907	0.43095240
H	7.03272669	1.87404010	1.91800996
H	4.96715753	0.91312808	2.82829491
H	-6.38387992	-0.65725003	2.48405712
H	-5.34516716	-2.07675555	2.17562407
H	-7.67906781	-2.54681398	2.29691468
H	7.19083845	-0.55220655	-2.44585419
H	6.18721845	-1.96945759	-2.03144435
H	8.42443677	-2.48276092	-1.80690262
H	-6.13604722	1.78329271	0.61791395
H	8.39116364	1.89130269	-0.51846369
H	0.17187144	2.58055211	2.50951213
H	-0.77734208	1.29198330	1.78414159

E = -2191.2475

14

C	0.10943475	-1.64599313	0.64283276
N	-0.10440214	-2.64422483	-0.31390812
B	-1.36907310	-2.79090952	-1.20592108
N	-2.27896860	-1.57112188	-0.89042299
C	-2.00128448	-0.56003989	0.03384340
C	-0.82791818	-0.61357989	0.80820923
C	1.37279719	-1.91086849	1.27772215
C	1.88158958	-3.06539802	0.67357337
C	0.93599255	-3.48672188	-0.30171015
C	-3.43662363	-1.29349989	-1.50420137
C	-3.95612473	-0.06683793	-1.01210269
C	-3.06965923	0.39820950	-0.03364101
C	-0.59514227	0.43498284	1.85173582
C	0.19280717	1.57095686	1.61568333
C	0.36091080	2.50290107	2.64638611
C	-0.23221014	2.31961507	3.88901951
C	-1.01328298	1.18824800	4.12180483
C	-1.19356936	0.25741603	3.10603514
F	-2.02743477	-3.98754440	-0.90174758
F	-1.01476739	-2.76847547	-2.56092660
C	2.00957750	-1.09455255	2.36440480
C	1.01656437	-4.68633697	-1.18729603
C	-3.20547491	1.65202956	0.78104729
C	-4.03202298	-2.18098479	-2.54768158
C	-5.26869842	0.47141652	-1.55170219
C	3.18972145	-3.82385162	0.82738108
O	3.99240161	-3.68493935	-0.35909408
C	4.73155549	-2.47028654	-0.40601815
C	5.79145001	-2.46382271	0.69949867
C	5.21112332	-2.98315412	1.98968786
C	4.01070223	-3.55123105	2.06027528
O	-6.38492981	-0.29422916	-1.04778102
C	-6.93868983	0.23658737	0.15594578
C	-7.60181883	1.57784504	-0.18359622
C	-6.58045816	2.46333875	-0.83535782
C	-5.50209081	1.95721927	-1.42944598
C	5.31656450	-2.31259324	-1.79679753
O	6.33064723	-3.29197935	-1.99706903
C	-7.90096087	-0.77885292	0.76331498

16

C	1.87216932	-2.59640567	-0.61248522
N	2.45270536	-3.23561673	0.48673226
B	3.70621723	-2.74288733	1.23871987
N	4.39015297	-1.68134217	0.34633981
C	3.78008279	-1.06291766	-0.75488890
C	2.53384011	-1.51589255	-1.22304860
C	0.65012854	-3.28660928	-0.92045372
C	0.55641123	-4.34807674	-0.01288471
C	1.67956334	-4.26875390	0.85140601
C	5.58680061	-1.11003786	0.55876774
C	5.78783293	-0.06710609	-0.38409538
C	4.67539027	-0.04461466	-1.23113241
C	1.94341639	-0.89715141	-2.45308099
C	1.15960682	0.26735315	-2.42221675
C	0.66779205	0.77463548	-3.63073176
C	0.94073826	0.15326583	-4.84351453
C	1.72088671	-1.00104665	-4.87014267
C	2.21436195	-1.51896783	-3.67939574
F	3.28690245	-2.13510595	2.46403996
F	4.55755057	-3.80595381	1.52155569
C	-0.34591807	-2.92114912	-1.98117467
C	2.00346045	-5.15377102	2.01040814
C	4.47702114	0.84297792	-2.42589858
C	6.53199441	-1.53091724	1.63345672
C	7.06282836	0.75594443	-0.33384564
C	-0.46301181	-5.45669857	0.18272261
O	-1.16076572	-5.28901167	1.43153103
C	-2.36793132	-4.53549258	1.33240083
C	-3.37855124	-5.35294109	0.51299535
C	-2.73514633	-5.74688863	-0.78716819
C	-1.41434946	-5.74508911	-0.95070572
O	8.19004559	0.02539410	-0.84350087
C	8.33732922	0.10136136	-2.25645806
C	8.68792735	1.54064692	-2.65082784
C	7.79329151	2.51234323	-1.92215798
C	7.00641325	2.14348008	-0.91549933
C	-2.81820226	-4.26185996	2.76513133
O	-4.05344892	-3.54484606	2.80311022
C	9.40238659	-0.88985352	-2.67536553
O	8.84648870	-2.19687106	-2.61509802
O	-4.56987096	-4.62855314	0.22224029
O	8.50774985	1.63182295	-4.07008548
C	0.81765951	0.97297027	-1.11282852
N	0.96538293	2.42218968	-1.15274897

O	-9.23115108	-0.52550749	0.30162360
O	6.21668531	-1.09615307	0.83839925
O	-8.12228630	2.21928176	0.97874833
C	0.88402936	1.81869379	0.29659997
O	2.28592850	1.97268350	0.54877452
C	3.06442341	2.16185695	-0.62163427
O	2.77734933	3.39782338	-1.25370602
C	3.49882122	4.51348191	-0.70771534
C	4.98493053	4.29806191	-1.00865545
C	5.39392487	2.97955464	-0.41959175
C	4.51121670	2.00252651	-0.23002318
O	5.78779975	5.34202179	-0.46599894
C	2.94121951	5.80622766	-1.28915205
O	3.67020934	6.16178545	-2.46688553
H	0.98405977	3.37242962	2.46681286
H	-0.08449354	3.05363294	4.67455678
H	-1.47972905	1.03203621	5.08911200
H	-1.79925899	-0.62629971	3.27902097
H	2.31871385	-0.11299869	1.99867013
H	1.32287061	-0.92212200	3.19494209
H	2.89664987	-1.58573875	2.75194877
H	2.05563309	-4.95603995	-1.37057718
H	0.50735680	-5.53964419	-0.72593293
H	0.52602103	-4.49658444	-2.14057419
H	-4.09973071	2.20236782	0.50205758
H	-3.27092775	1.43561513	1.84917077
H	-2.34768617	2.31675123	0.65421038
H	-3.79131798	-3.22427401	-2.34905310
H	-5.11569982	-2.06854542	-2.57146106
H	-3.63309087	-1.94055861	-3.53892855
H	-5.28171114	0.24648897	-2.62436243
H	2.94715440	-4.89257272	0.83745052
H	4.06020274	-1.61701833	-0.22971686
H	6.63674235	-3.08343969	0.38086472
H	5.81098374	-2.84019519	2.88632509
H	3.59654963	-3.87949573	3.01004360
H	-6.13608114	0.41811126	0.88331404
H	-8.41414702	1.37581918	-0.89100669
H	-6.76340749	3.53322638	-0.79480485
H	-4.76113153	2.60072020	-1.89608979
H	5.72471256	-1.29748045	-1.88297889
H	4.50296657	-2.42991866	-2.52363191
H	6.64552616	-3.22251466	-2.90375566
H	-7.86353624	-0.69624492	1.85678059
H	-7.56277936	-1.78074804	0.47807264
H	-9.79974007	-1.24464896	0.59495751
H	7.08288322	-1.08130749	1.25861999
H	-8.95258593	1.77076592	1.18049118
H	0.49914231	2.72986626	-0.17598916
H	0.72092921	0.99032254	-0.40051324
H	2.77432550	1.40906629	-1.36651904
H	3.36395007	4.53504383	0.38010087
H	5.10318044	4.26464297	-2.09828273
H	6.44164444	2.86209441	-0.15910175
H	4.79840017	1.05132546	0.20669417
H	5.69465661	6.09008754	-1.06845709
H	3.03212171	6.60084027	-0.53811413
H	1.87844944	5.65480867	-1.50879814
H	3.23881748	6.91679914	-2.87973664

E = -2562.0451

C	0.04479567	3.41268758	-1.39610602
O	0.36654610	4.59607993	-1.40567707
N	-1.25174810	3.01619802	-1.64426034
C	-2.28671055	4.01020732	-1.87750533
O	5.68186206	3.15142966	2.38341626
C	4.25954763	3.21388653	2.47388644
O	-11.33878241	-0.43837106	-1.68818927
C	-10.17308357	0.33418319	-1.42015971
C	1.66909251	1.71637010	4.52814498
C	2.84835160	1.22804678	4.90624005
C	4.05261695	1.16546376	4.00102420
C	3.76189623	1.77072639	2.60659214
O	2.38815609	1.66479383	2.21391811
C	-6.95323834	-0.13772342	-2.46985911
C	-7.65815393	-1.24350022	-2.69351457
C	-8.69873188	-1.73058216	-1.71787522
C	-9.13947128	-0.59927512	-0.78874171
O	-8.02340053	0.13660828	-0.27869078
C	-7.14165778	0.72213426	-1.24222222
C	1.45379396	2.25975619	3.13519898
C	-0.08196464	-0.53125333	2.75110751
C	-0.44006035	4.53873283	2.01872785
C	-5.13452907	-1.44150243	-0.04413006
C	-5.89124263	3.59369492	-0.64410829
F	-2.06166843	-0.87745219	0.31368147
F	-3.15175235	-0.86177012	2.33902846
C	-3.62608261	5.21192136	1.53411327
C	-3.73061332	6.58050991	1.30902698
C	-3.32143855	7.10501897	0.08622670
C	-2.82882253	6.25491287	-0.89888574
C	-2.73774020	4.87402260	-0.70074971
C	-3.13398488	4.35435317	0.54482858
C	-0.75060880	3.07147633	2.04938017
C	0.07426980	2.07014376	2.57067404
C	-0.57293529	0.82667137	2.37553960
C	-4.97803119	0.03610464	0.00659608
C	-5.83947205	1.01999945	-0.54287616
C	-5.27547613	2.27095979	-0.29300966
C	-3.03525151	2.89457824	0.84777084
C	-1.91517148	2.41179982	1.54275768
N	-1.75801926	1.03784568	1.76655541
B	-2.71928856	-0.05895329	1.25347445
N	-3.92103022	0.64519458	0.57499993
C	-4.04250379	2.02972622	0.39701601
O	-8.20475894	-2.78300989	-0.87266946
O	4.58805298	-0.15160448	3.94955232
H	0.06744138	1.67767599	-3.61783582
H	0.54901746	0.57081533	-5.76557695
H	1.94728443	-1.49227450	-5.81084886
H	2.83046915	-2.41226585	-3.68928873
H	-0.62552865	-1.86934969	-1.91499082
H	0.04480157	-3.08621372	-2.98872085
H	-1.25579558	-3.50514861	-1.87113505
H	1.11980869	-5.70246166	2.32990197
H	2.79547513	-5.86330625	1.75258884
H	2.36998990	-4.56759487	2.85372034
H	5.36854976	1.42987760	-2.62705899
H	4.25194675	0.26296796	-3.32209726
H	3.64415133	1.53864994	-2.29435645
H	6.39355863	-2.57945710	1.88559992
H	7.55935431	-1.37927926	1.30186328
H	6.35798897	-0.94494591	2.54254880
H	7.30350145	0.88665631	0.72634013
H	0.10923551	-6.37902695	0.34242690
H	-2.18861008	-3.58154490	0.81864105
H	-3.62555131	-6.26085135	1.08903382
H	-3.40750007	-6.00768914	-1.59975569
H	-0.96520756	-6.00837305	-1.90452545
H	7.39993406	-0.17178292	-2.75824859
H	9.74175447	1.74038321	-2.39848901
H	7.78850928	3.53939241	-2.28414474
H	6.33845239	2.84410084	-0.42304395
H	-2.02861637	-3.72053169	3.29567234
H	-2.98240134	-5.21023713	3.28334493
H	-3.85794041	-2.59906327	2.73625968
H	10.25960992	-0.78694620	-1.99507264

			H 9.72451689 -0.63871197 -3.69405155
			H 9.55041964 -2.83565270 -2.76665855
			H -4.87223611 -4.25083173 1.06338264
			H 8.82254597 2.49350877 -4.36519595
			H -0.19772473 0.70674925 -0.80521658
			H 1.45357789 0.60935492 -0.30513680
			H 1.87292277 2.79639751 -0.92618973
			H -1.53732194 2.07046789 -1.45524380
			H -1.93995054 4.67880674 -2.66679648
			H -3.14850073 3.46777819 -2.27759873
			H 6.04489993 4.03604721 2.49617839
			H 3.95370288 3.82276267 3.33565457
			H 3.82315690 3.65479161 1.56861174
			H -12.01035274 0.13574378 -2.07035118
			H -9.78187701 0.78474001 -2.34324535
			H -10.37960166 1.13951634 -0.70346988
			H 0.83011316 1.76236073 5.21719535
			H 2.99201671 0.82474100 5.90507326
			H 4.86280737 1.75163271 4.44635343
			H 4.30370736 1.17912273 1.86730325
			H -6.21481271 0.21480150 -3.18575268
			H -7.49010004 -1.82989361 -3.59448880
			H -9.58148378 -2.08912969 -2.25971884
			H -9.59202843 -1.05508373 0.09378594
			H -7.56541884 1.67853511 -1.58568239
			H 1.64130112 3.34083243 3.17067659
			H -0.33730673 -1.26602822 1.98796221
			H 0.99723066 -0.52677576 2.88311750
			H -0.54185954 -0.86118175 3.68861446
			H 0.55575637 4.73326400 2.41681657
			H -0.46238118 4.94126953 1.00525610
			H -1.14778442 5.11805525 2.61510568
			H -6.18571389 -1.72157052 -0.05498427
			H -4.67160041 -1.84847690 -0.94847243
			H -4.64515498 -1.92258942 0.79494447
			H -5.81498734 4.30886578 0.17415253
			H -5.41670663 4.06050931 -1.51259840
			H -6.94976620 3.47475116 -0.87886450
			H -3.93131834 4.79264817 2.48776243
			H -4.11846576 7.23071280 2.08632741
			H -3.37783957 8.17277758 -0.09897752
			H -2.49390277 6.67044115 -1.84422606
			H -7.64743200 -3.37834764 -1.38818783
			H 3.96057641 -0.76225555 3.53179972
			E = -4278.1908

3. Copies of ^{1}H NMR and ^{13}C NMR spectra

^{1}H NMR 300 MHz, $CDCl_3$ **9**

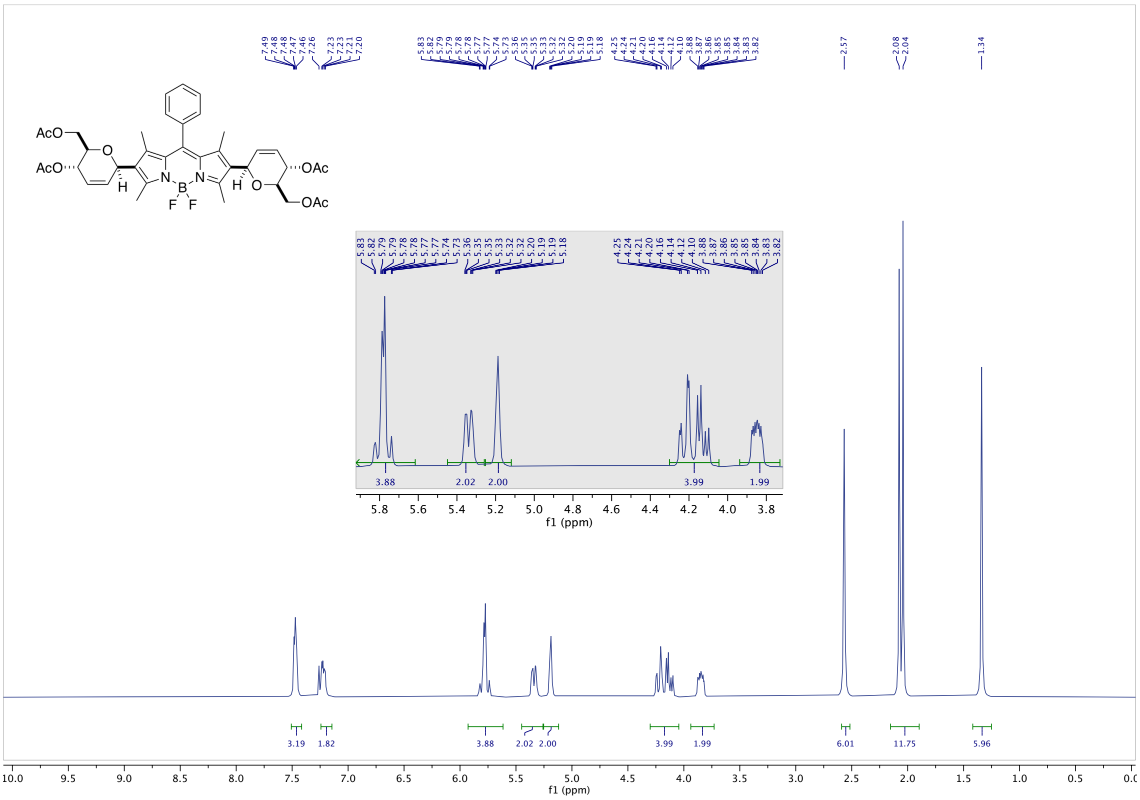

Figure S5. ^{1}H NMR for compound **9** (300 MHz, $CDCl_3$)

^{13}C{^{1}H} NMR 75 MHz, $CDCl_3$, **9**

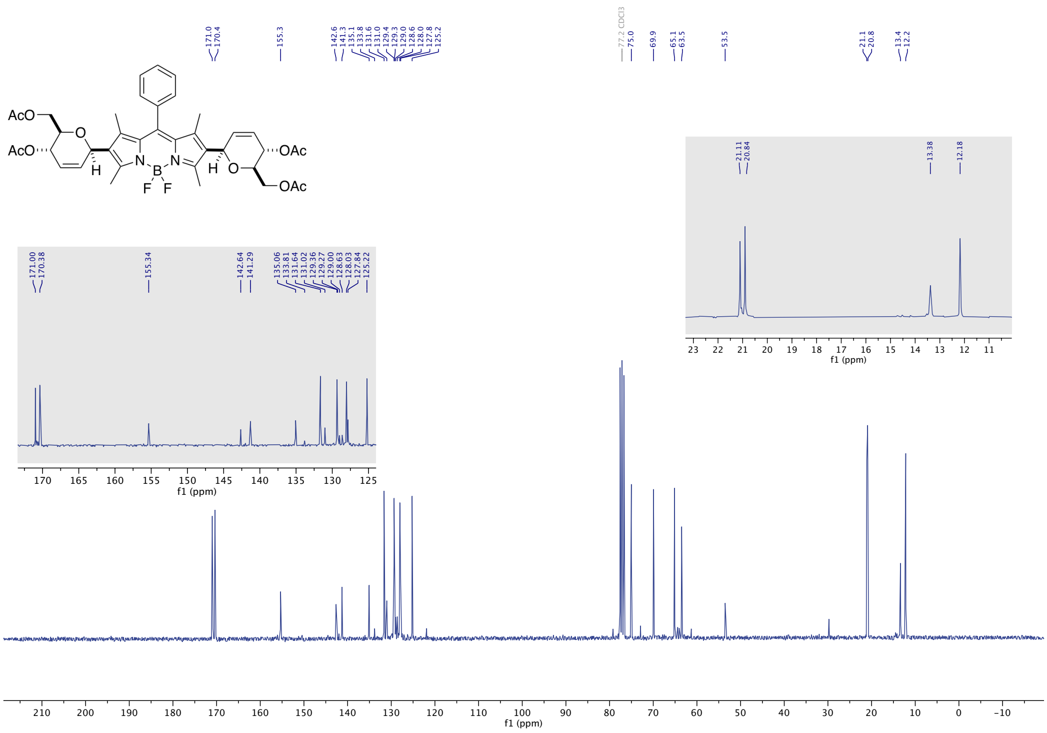

Figure S6. ^{13}C{^{1}H} NMR for compound **9** (75 MHz, $CDCl_3$)

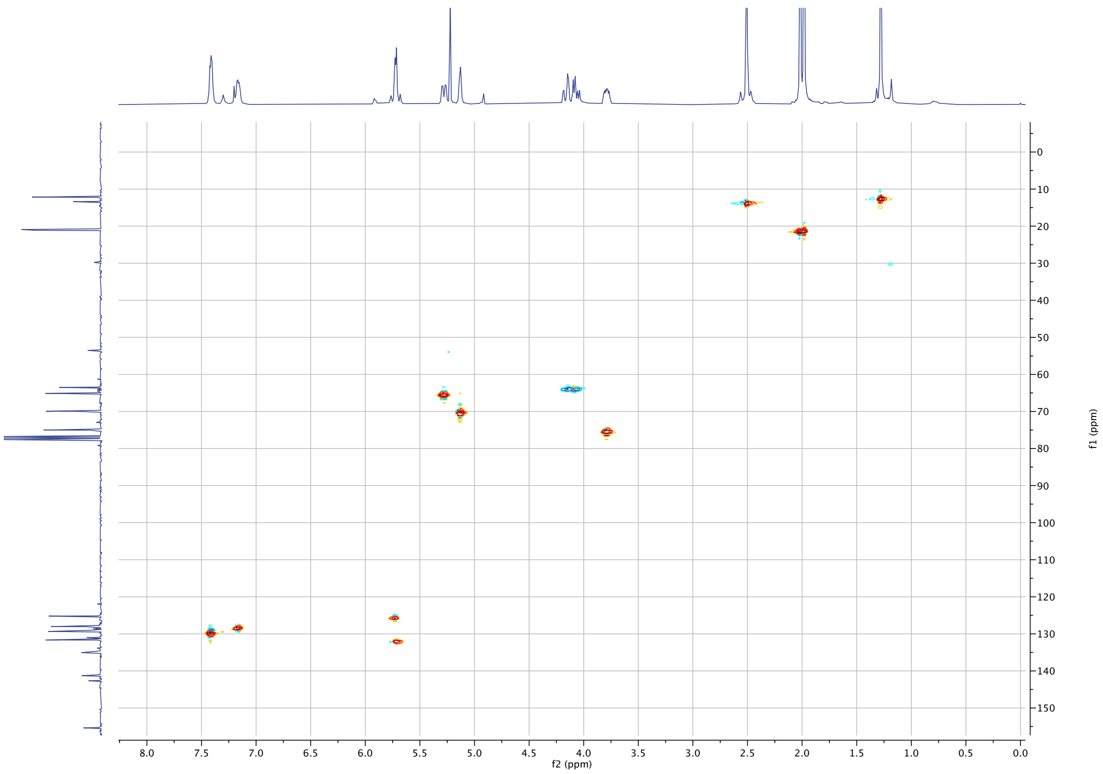

Figure S7. ^{1}H-^{13}C HSQC for compound **9**

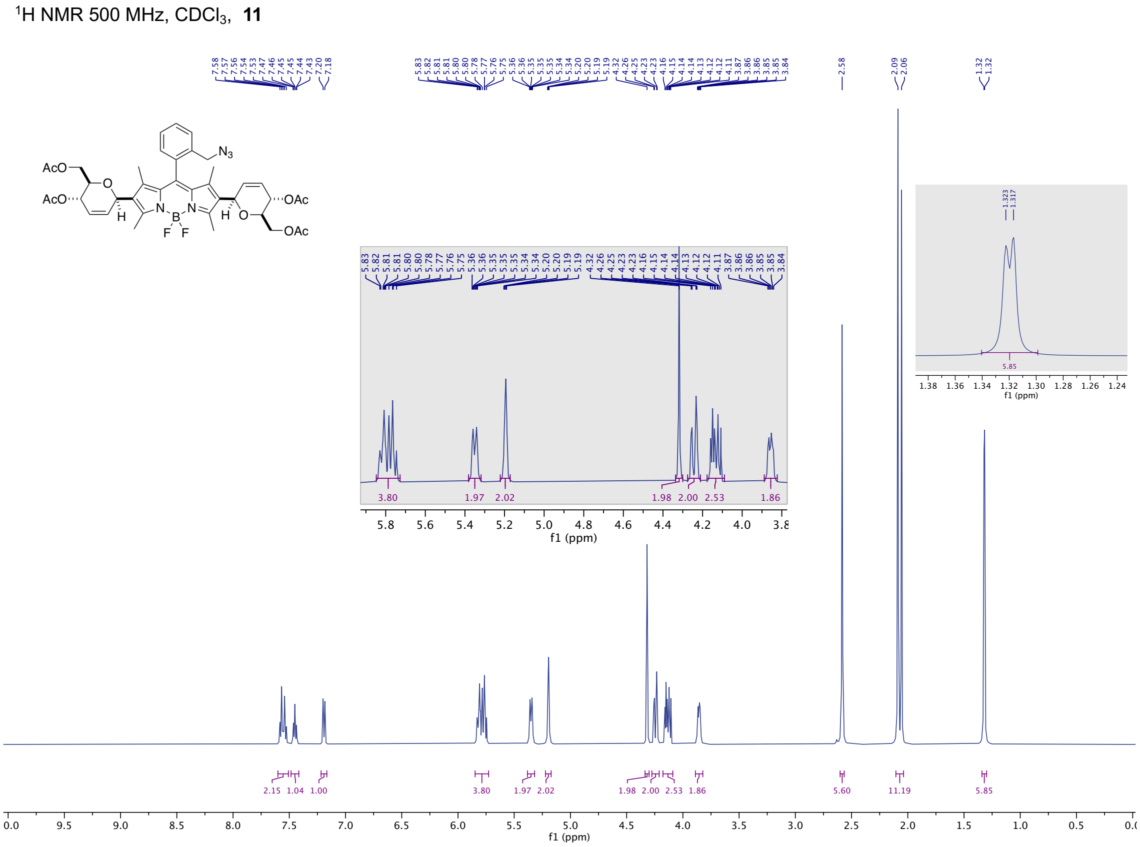

Figure S8. ^{1}H NMR for compound **11** (500 MHz, $CDCl_3$)

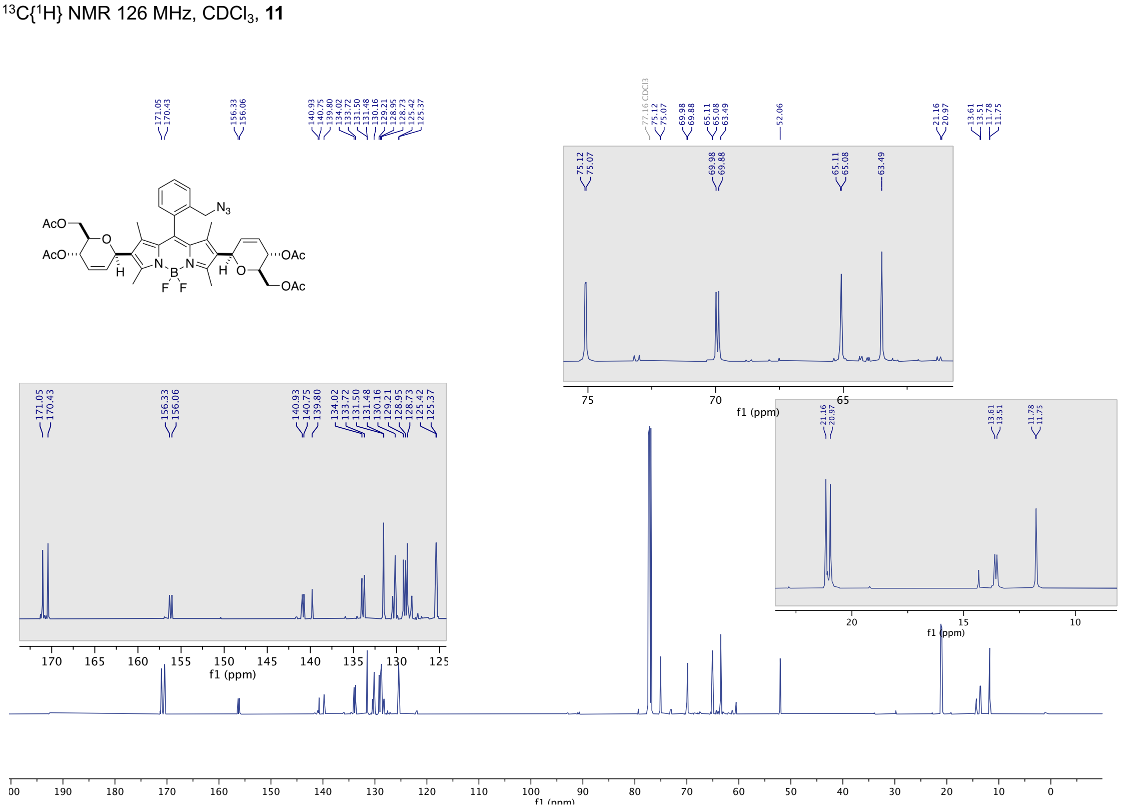

Figure S9. $^{13}C\{^1H\}$ NMR for compound **11** (126 MHz, $CDCl_3$)

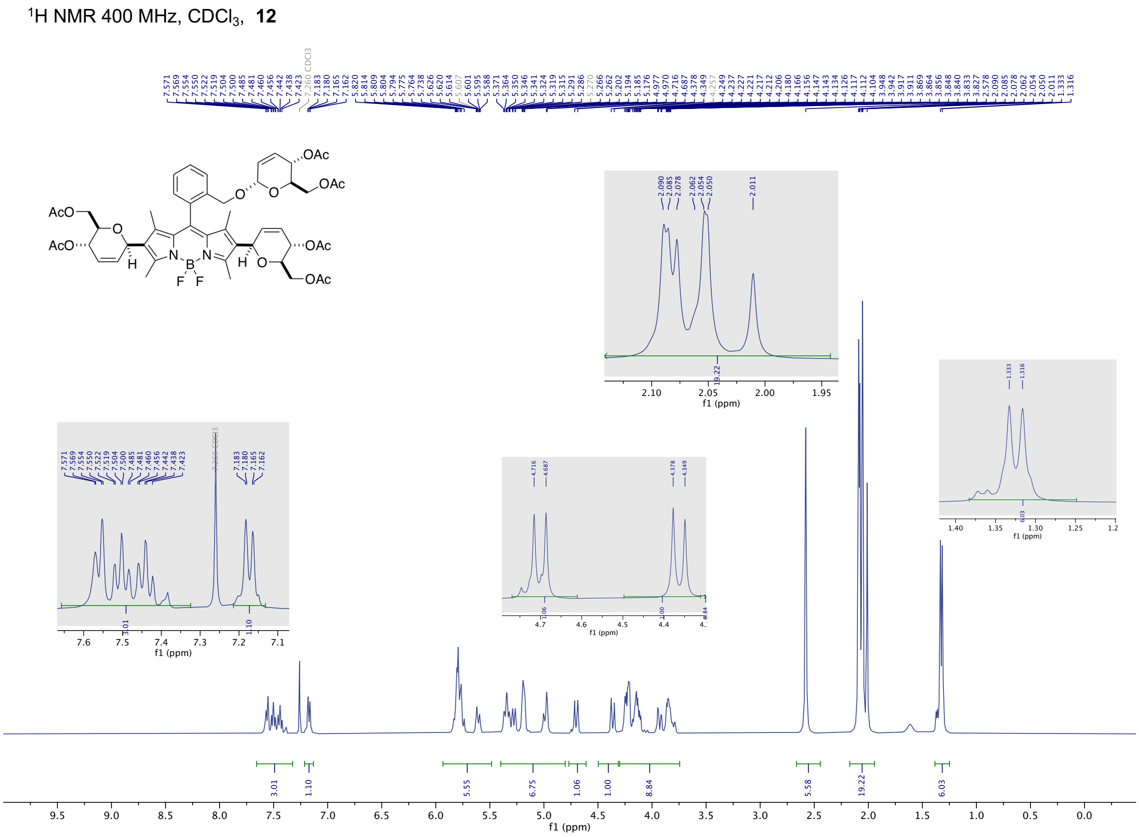

Figure S10. ^{1}H NMR for compound **12** (400 MHz, CDCl$_3$)

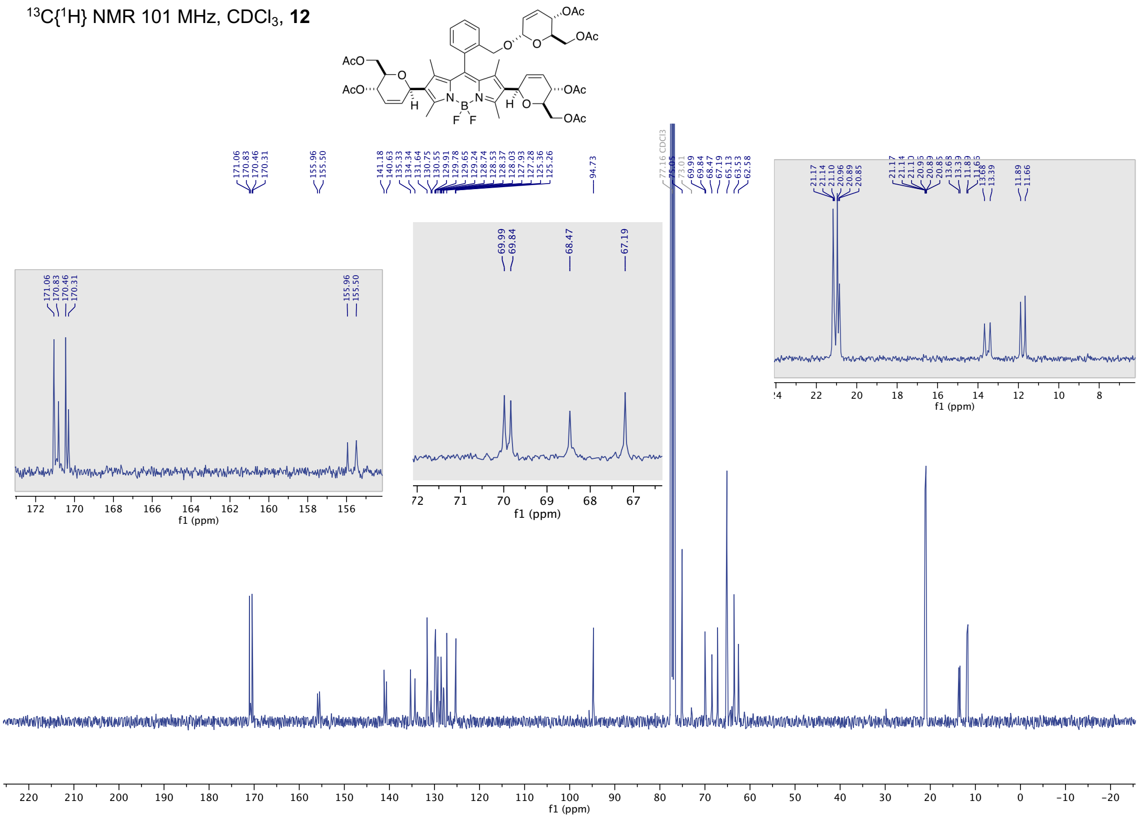

Figure S11. ^{13}C{^{1}H} NMR for compound **12** (101 MHz, CDCl$_3$)

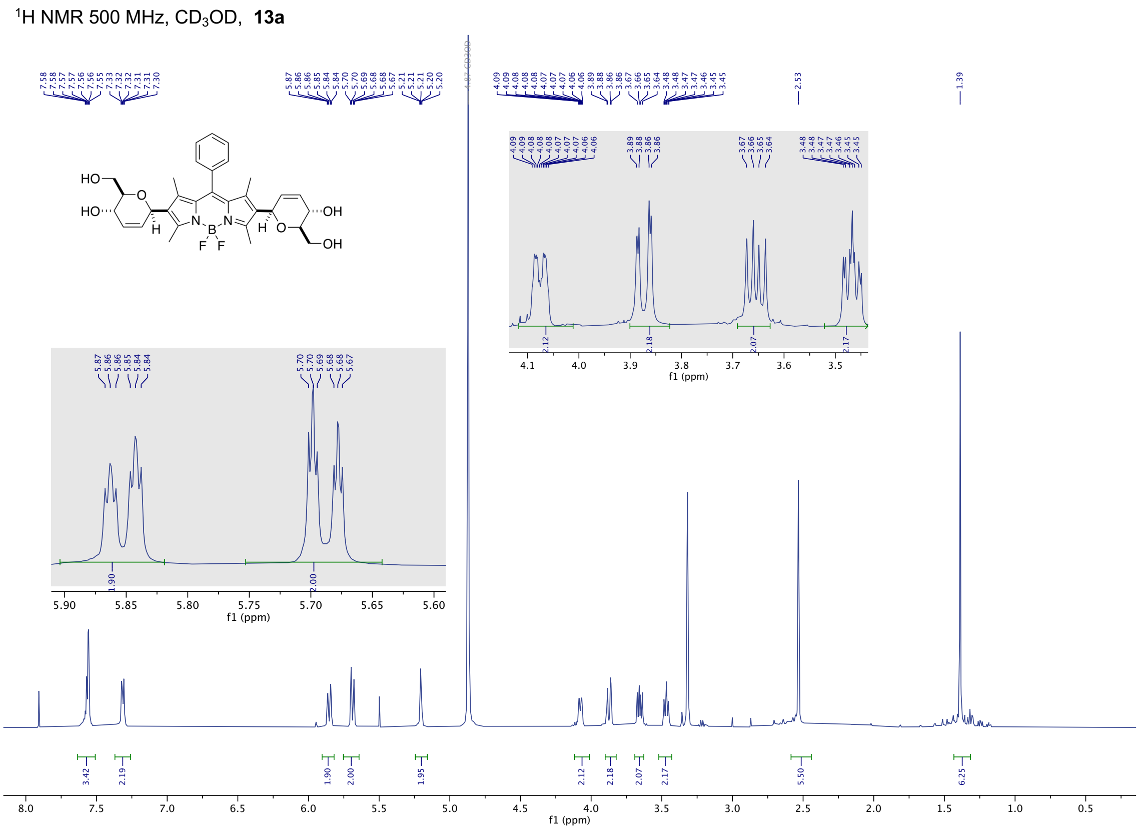

Figure S12. ^{1}H NMR for compound **13a** (500 MHz, CD$_3$OD)

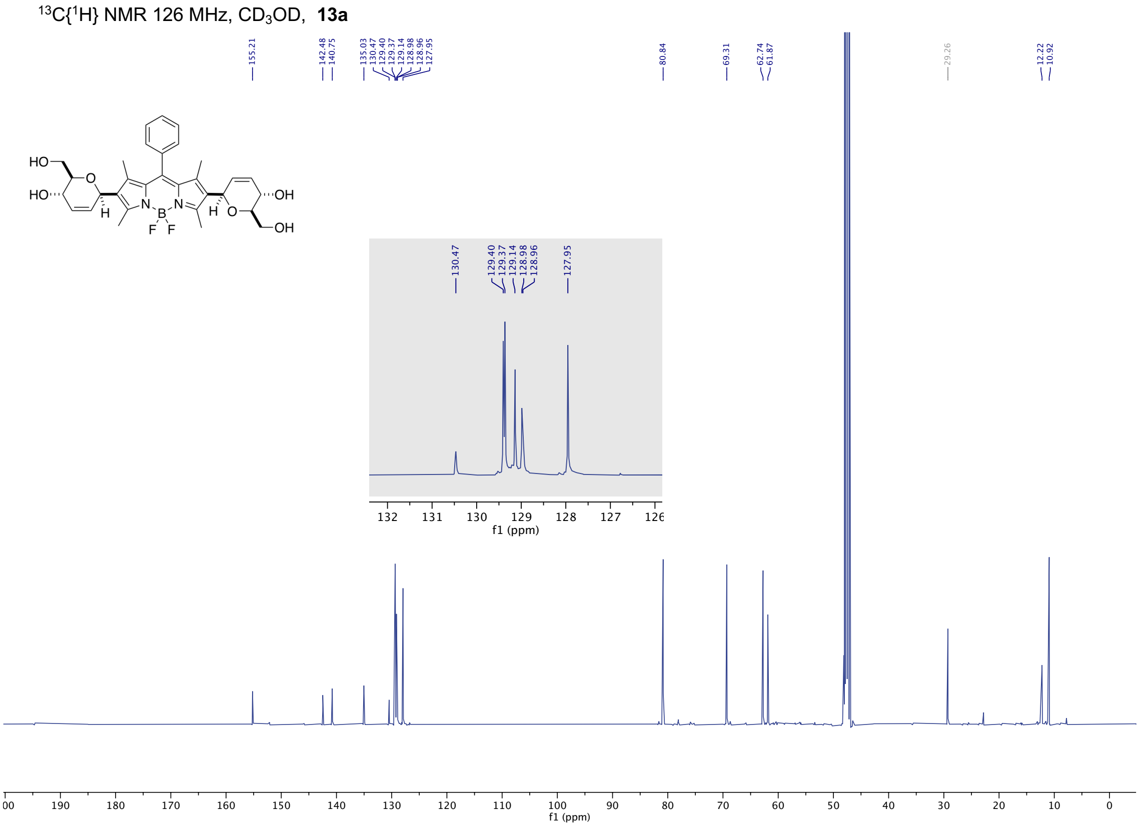

Figure S13. ^{13}C{^{1}H} NMR for compound **13a** (126 MHz, CD$_3$OD)

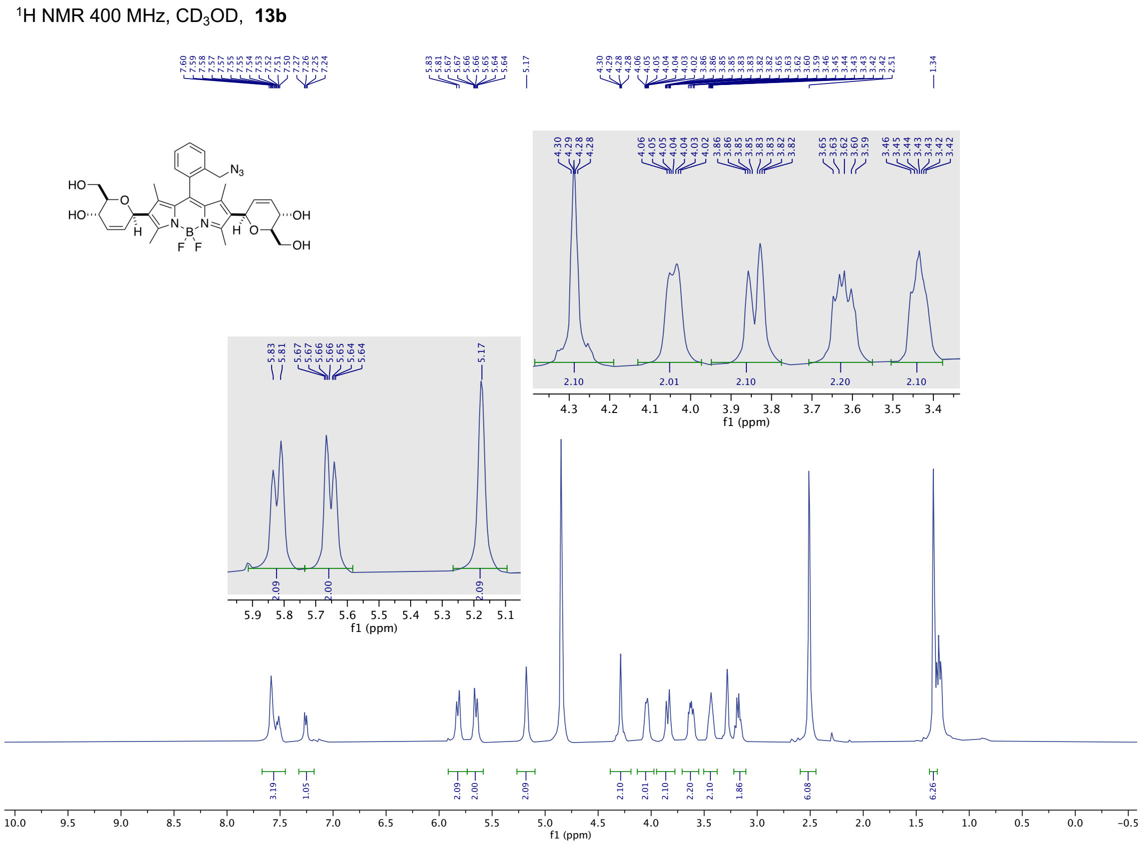

Figure S14. ^{1}H NMR for compound **13b** (400 MHz, CD_3OD)

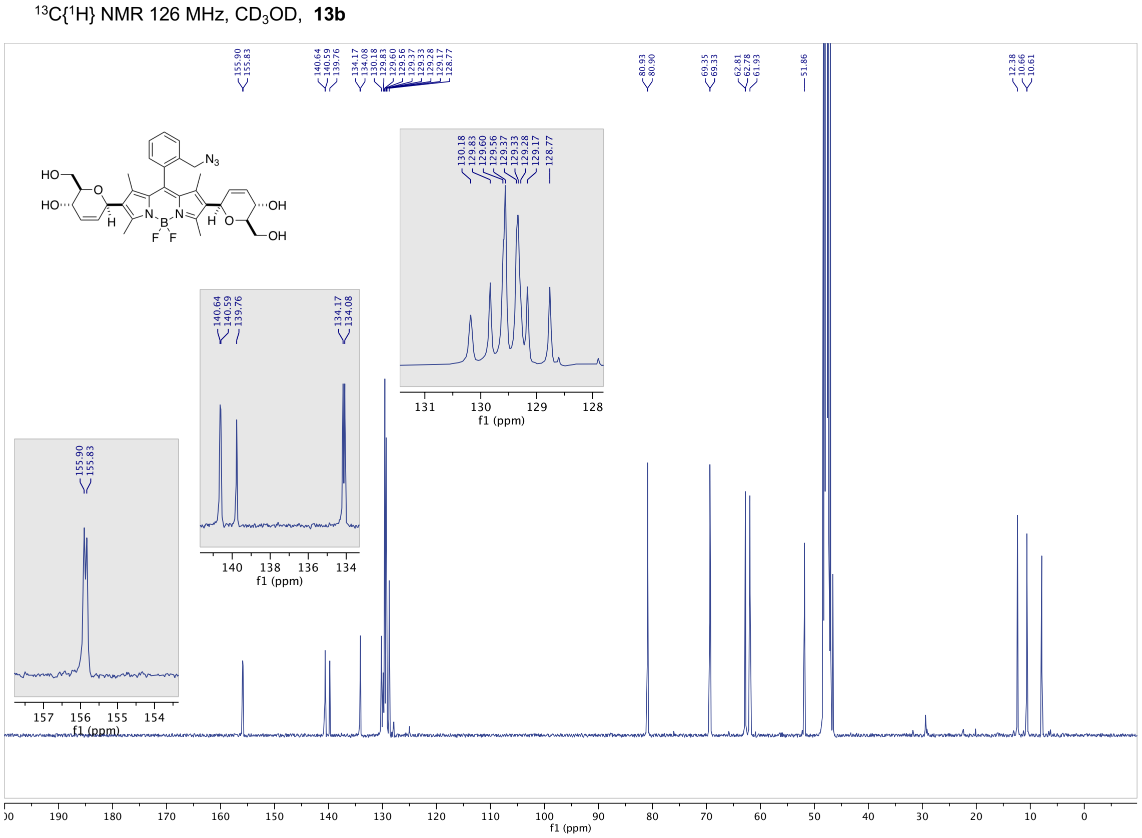

Figure S15. ^{13}C{^{1}H} NMR for compound **13b** (126 MHz, CD_3OD)

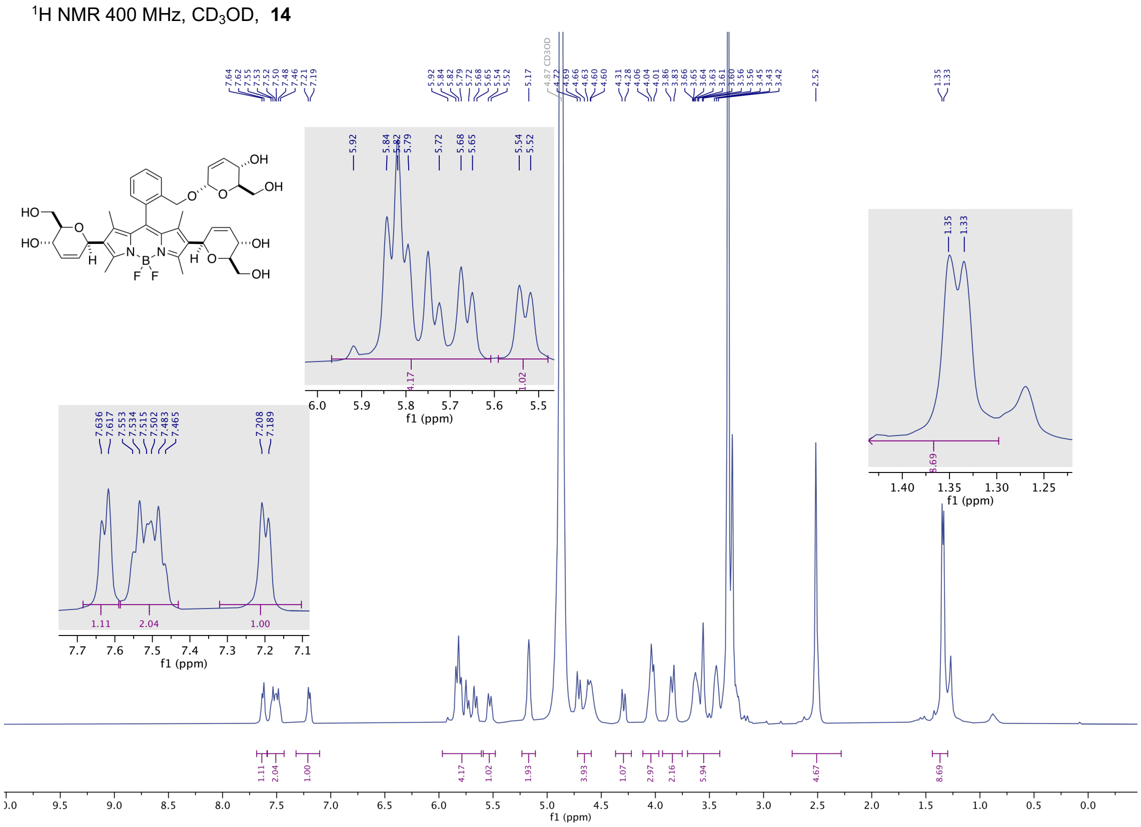

Figure S16. ^{1}H NMR for compound **14** (400 MHz, CD_3OD)

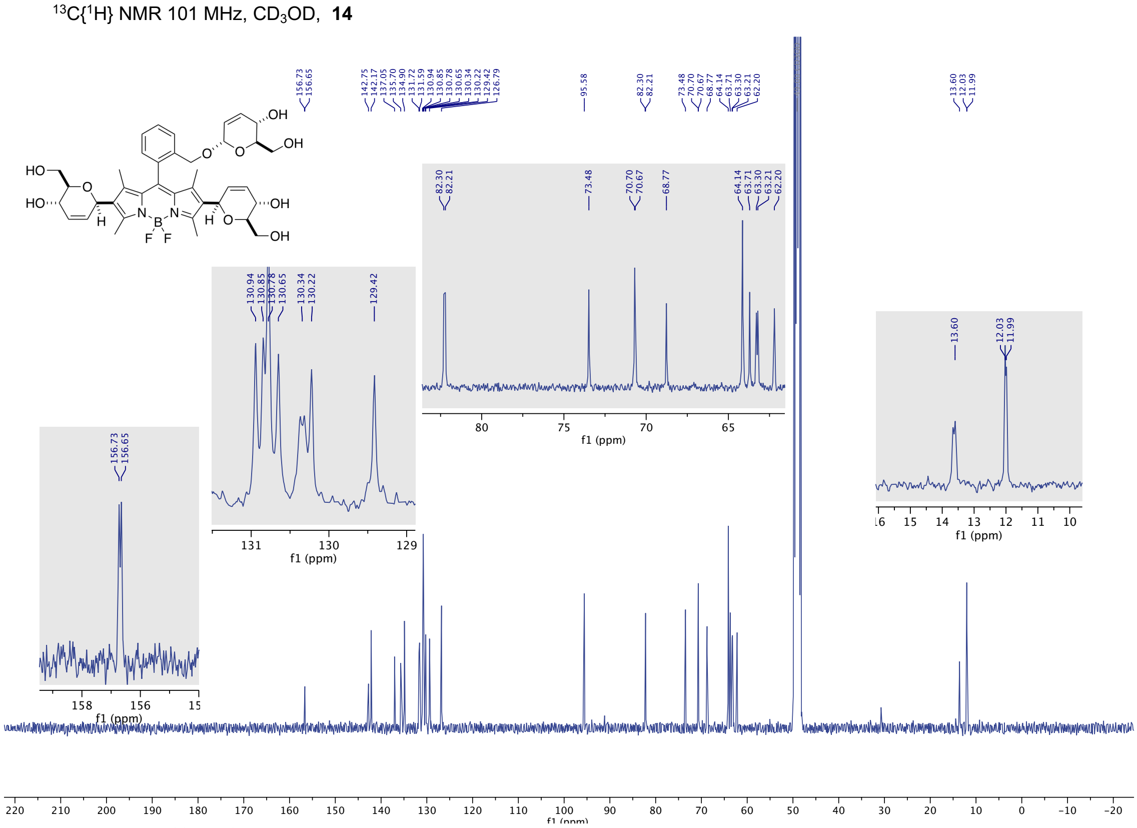

Figure S17. $^{13}C\{^{1}H\}$NMR for compound **14** (101 MHz, CD_3OD)

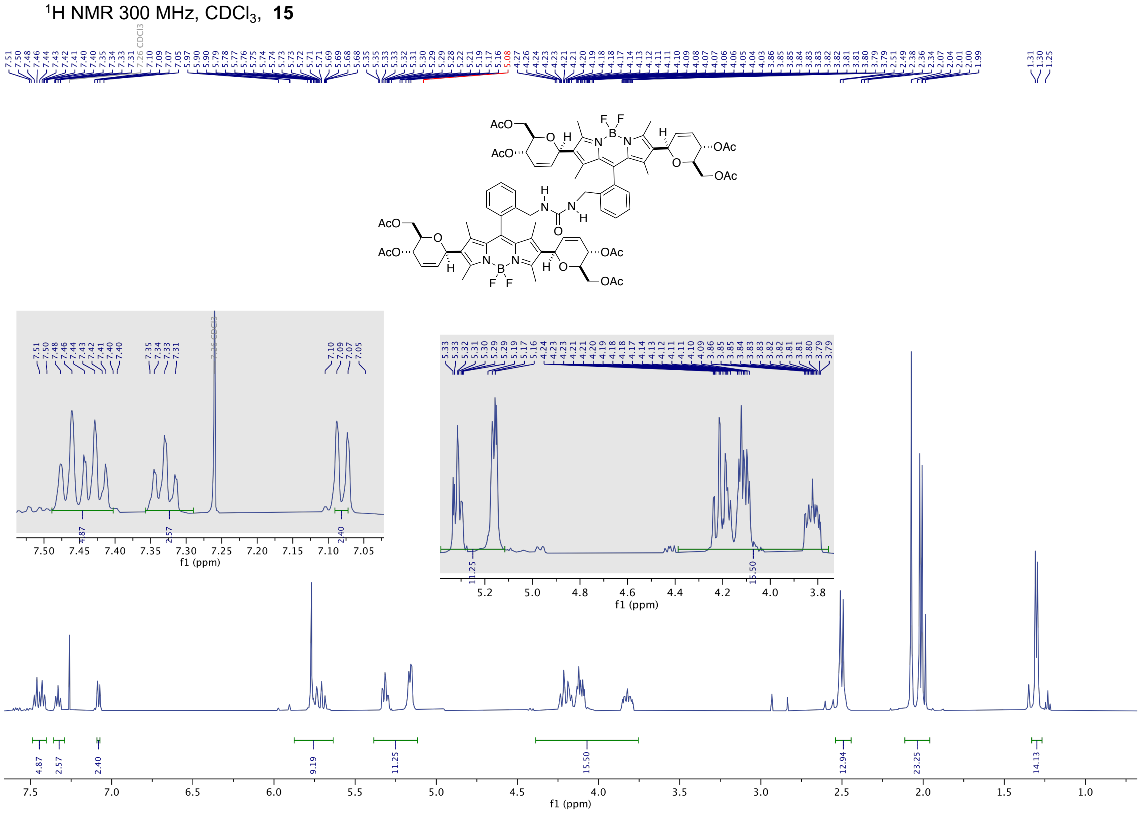

Figure S18. ^{1}H NMR for compound **15** (300 MHz, $CDCl_3$)

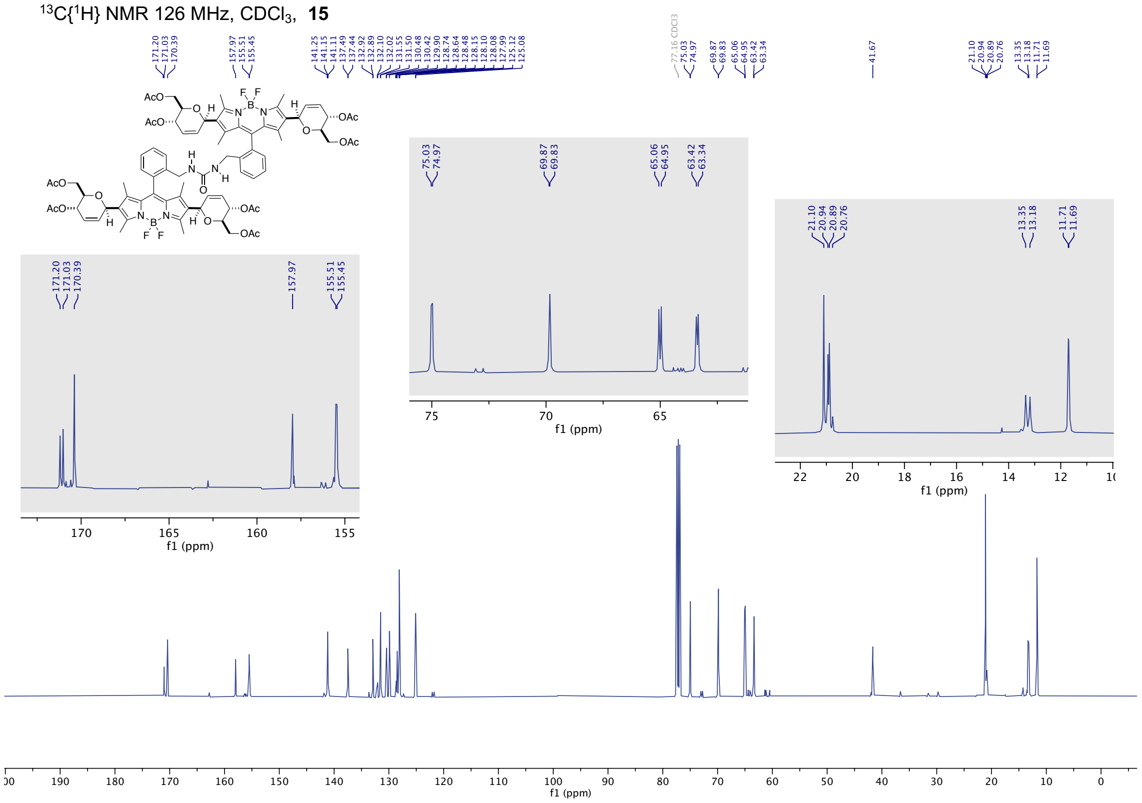

Figure S19. $^{13}C\{^{1}H\}$ NMR for compound **15** (126 MHz, $CDCl_3$)

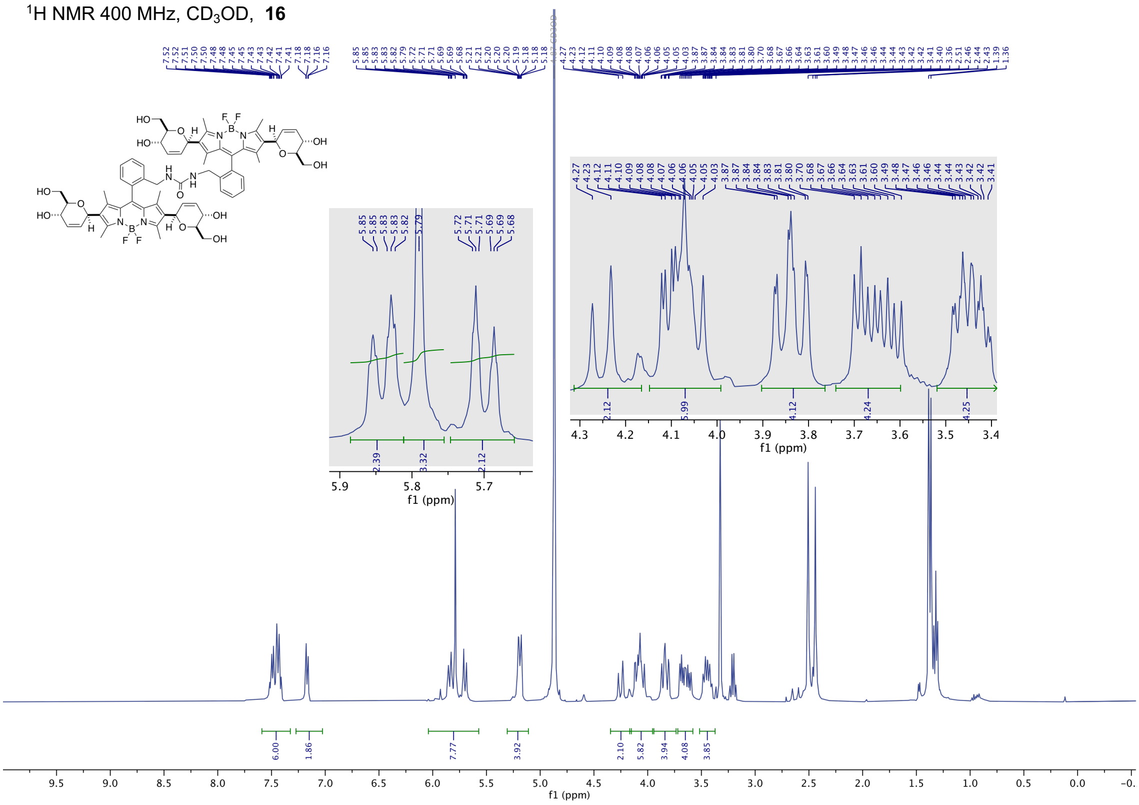

Figure S20. ^{13}C NMR for compound **15** (400 MHz, CD$_3$OD)

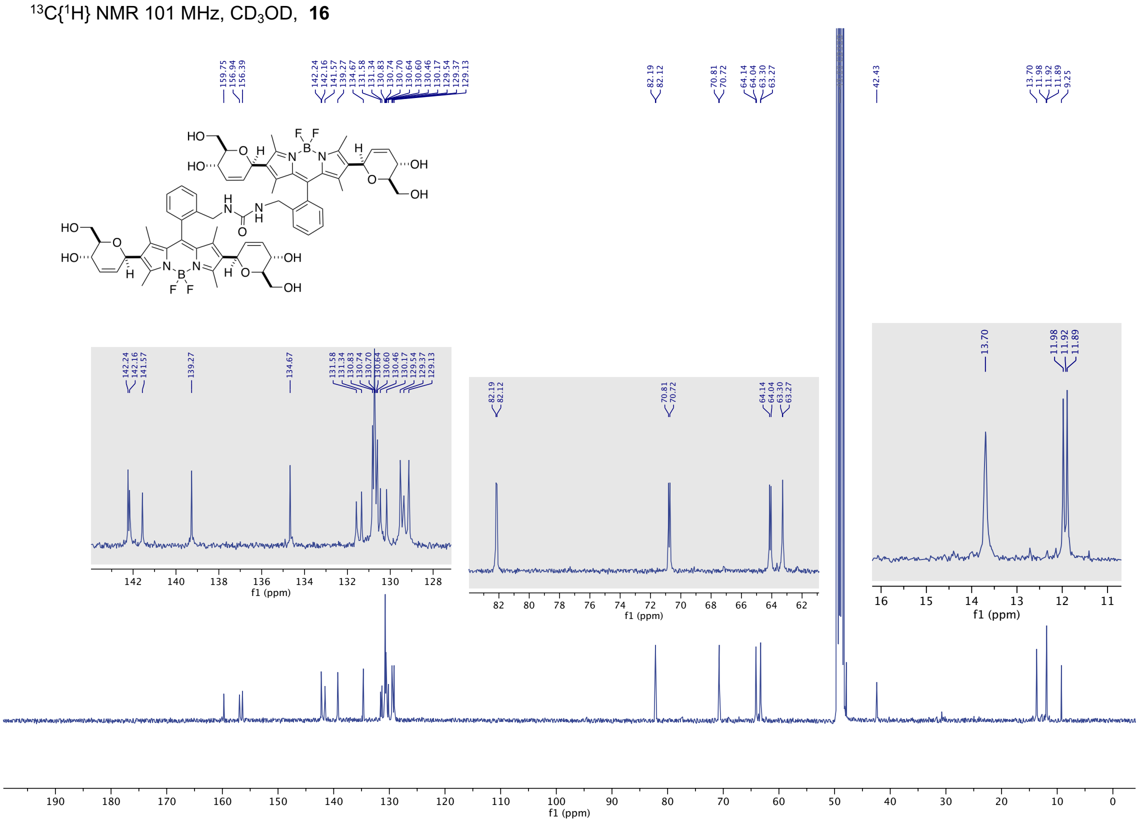

Figure S21. ^{13}C{^{1}H} NMR for compound **16** (101 MHz, CD$_3$OD)

^{1}H NMR 300 MHz, CDCl$_3$, **10**

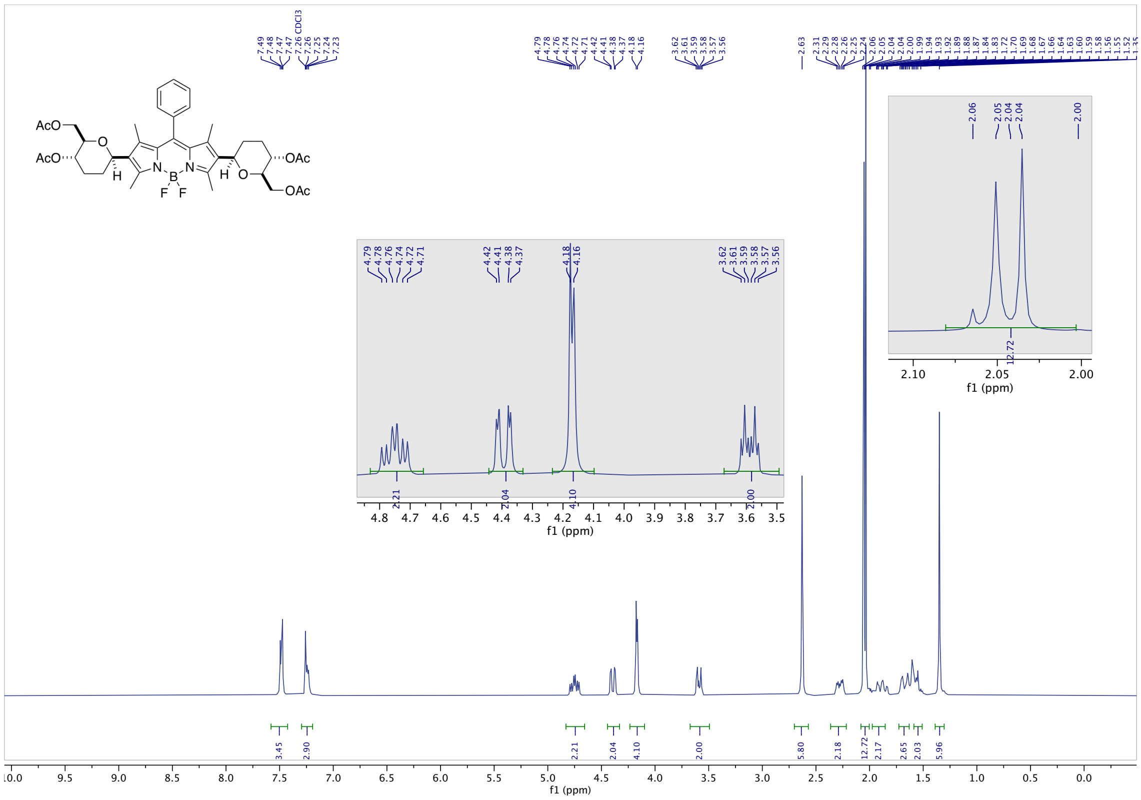

Figure S22. ^{1}H NMR for compound **10** (300 MHz, CDCl$_3$)

^{13}C{^{1}H} NMR 101 MHz, CDCl$_3$, **10**

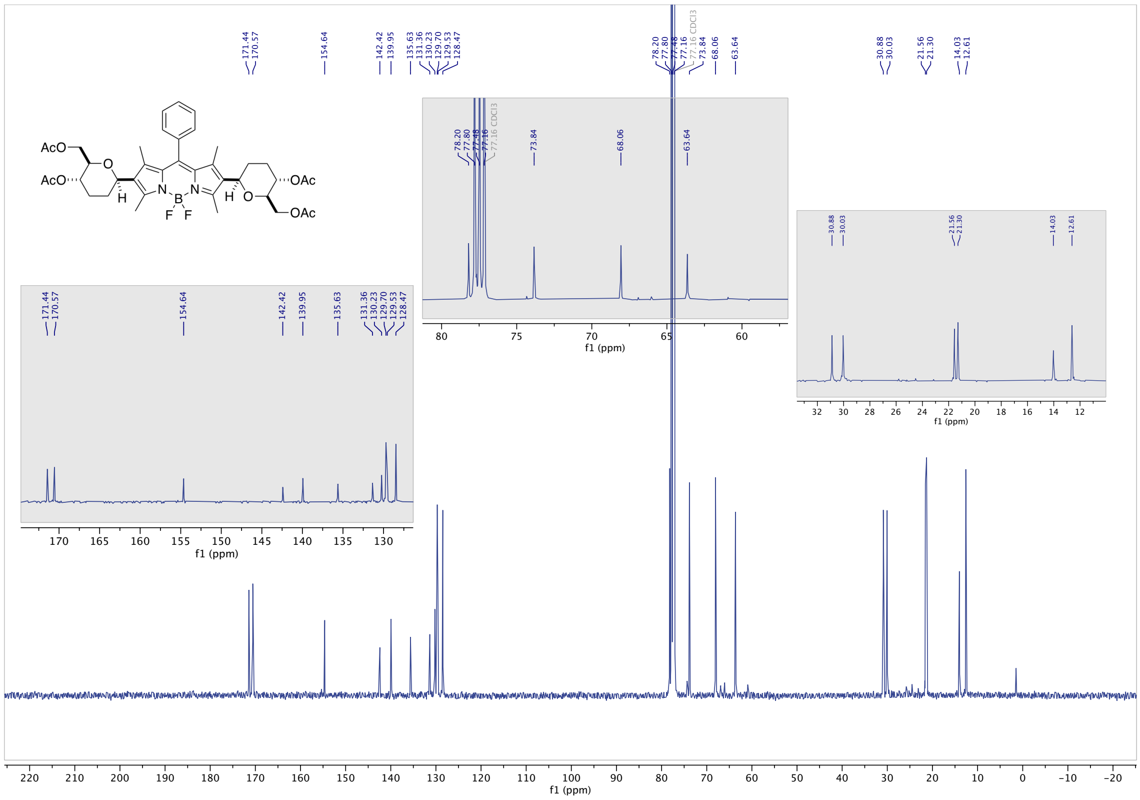

Figure S23. ^{13}C{^{1}H}NMR for compound **10** (101 MHz, CDCl$_3$)
